# Dynamics of the Ligand Excited States Relaxation in Novel β-Diketonates of Non-Luminescent Trivalent Metal Ions

**DOI:** 10.3390/ijms24098131

**Published:** 2023-05-01

**Authors:** Trofim Polikovskiy, Vladislav Korshunov, Victoria Gontcharenko, Mikhail Kiskin, Yuriy Belousov, Claudio Pettinari, Ilya Taydakov

**Affiliations:** 1 P. N. Lebedev Physical Institute of the Russian Academy of Sciences, 53 Leninskiy 1. Prospect, 119991 Moscow, Russia; 2 Faculty of Chemistry, National Research University Higher School of Economics, 20 Miasnitskaya Str., 101000 Moscow, Russia; 3 Kurnakov Institute of General and Inorganic Chemistry, Russian Academy of Sciences, 119991 Moscow, Russia; 4 Chemistry Department, M. V. Lomonoso sv Moscow State University, Leninskie Gory Str, Building 1/3, 119991 Moscow, Russia; 5 Chemistry Interdisciplinary Project (ChIP), School of Pharmacy, University of Camerino, Via Madonna delle Carceri, 62032 Camerino, Italy

**Keywords:** luminescence, lanthanides, rare earths, coordination compounds, 1,3-diketones, 4-acylpyrazolones, aluminum, gallium, indium

## Abstract

Complexes emitting in the blue spectral region are attractive materials for developing white-colored light sources. Here, we report the luminescence properties of novel coordination compounds based on the trivalent group 3, 13 metals, and the 1-phenyl-3-methyl-4-cyclohexylcarbonyl-pyrazol-5-onate (Q^CH^) ligand. [M(Q^CH^)_3_] (M = Al, Ga, and In), [M(Q^CH^)_3_(H_2_O)] (M = Sc, Gd, and Lu), [Lu(Q^CH^)_3_(DMSO)], and [La(Q^CH^)_3_(H_2_O)(EtOH)] complexes were synthesized and structurally characterized by a single-crystal X-ray diffraction study. It has been found that the luminescence quantum yields of the ligand increase by one order of magnitude upon metal coordination. A significant correspondence between the energies of the ligand’s excited states and the luminescence quantum yields to the metal ion’s atomic numbers was found using molecular spectroscopy techniques. The replacement of the central ion with the heavier one leads to a monotonic increase in singlet state energy, while the energy of the triplet state is similar for all the complexes. Time-resolved measurements allowed us to estimate the intersystem crossing (ISC) rate constants. It was shown that replacing the Al^3+^ ion with the heavier diamagnetic Ga^3+^ and In^3+^ ions decreased the ISC rate, while the replacement with the paramagnetic Gd^3+^ ion increased the ISC rate, which resulted in a remarkably bright and room-temperature phosphorescence of [Gd(Q^CH^)_3_(H_2_O)].

## 1. Introduction

White organic light-emitting diodes (WOLEDs) are the most economical light sources for street, home, and display lighting and many other special applications [1,2,3]. White emissions in such devices are conditioned by simultaneous emissions of several luminophores in blue, green, and red spectral areas [2], providing an R-G-B scheme of white light [3], or emissions of blue and orange emitters, providing a B-O scheme. One of the most popular classes of materials used in WOLEDs are the platinum-based group materials, especially those based on Ir(III) complexes containing 2-phenylpyridine fluorinated derivatives and imidazole-type carbene compounds [4,5]. The commercial price of production of such materials is relatively high, which makes the technology quite expensive [6].

An attractive class of blue light-emitting materials is formed by small organic molecules, such as triarylboranes and triarylmethanes [7,8]. A great interest has arisen in recent years for a wide range of such compounds and a variety of approaches for changing photophysical properties by small variations in the chemical structure has been reported [9]. Among these compounds, β-diketones are noteworthy molecules due to the possibility of fine-tuning the energy of first excited singlet (S_1_) and triplet (T_1_) states and their potential application in OLEDs [10,11]. Furthermore, phosphorescence or even thermally activated delayed fluorescence (TADF) can be achieved in some cases [12]. However, β-diketones often have critically low emission quantum yields due to several non-radiative relaxation processes, in particular, vibration multiphonon relaxation on O-H and C-H oscillators [13]. Keto-enol tautomerism makes the energy relaxation dynamic more complicated due to excited state intermolecular proton transfer (ESIPT), which is associated with proton localization near the diketone oxygen atom [14]. This limits the luminescence efficiency of diketone derivatives. Luckily, the luminescence performance of β-diketones can be dramatically improved by the formation of metal complexes. When β-diketonates are coordinated to metal, they exist exclusively in the enolic form and consequently, a significant increase in the luminescence quantum yield is observed [9,15].

On the other hand, luminescence efficiency can be adjusted by changing the energies of S_1_ and T_1_ as well as the excited states’ lifetimes [16,17,18]. There are many studies aimed at the investigation of the dependence of these photophysical properties on minor chemical structure change [19,20,21].

Notably, there is currently no general theory describing how photophysical properties of a ligand change upon coordination to different metal ions. Several empirically derived guidelines enable a prediction of the energy change in the first excited singlet state [22], the triplet state [23], or the luminescence quantum yield [24]. According to these rules, we can estimate the luminescence efficiency of coordination compounds. However, such rules are rather imprecise, and there are many exceptions to them [25]. In addition, no systematic study has been reported on the role of the metal ion in coordination compounds on the energies of excited ligand states, on the energy transfer processes, and, consequently, on the luminescence quantum yield.

Among all the β-diketonate ligands, 4-acylpyrazolonates must be highlighted due to their outstanding properties [26,27,28], such as their sufficient chemical and thermal stability, comparably effortless chemical synthesis, and relatively high energy of the first excited triplet state T_1_, which can range from 21,000 to 24,000 cm^−1^ depending on the ligand substituents [29,30,31,32,33,34,35,36].

The spectroscopic characteristics of β-diketonate complexes, i.e., the luminescence quantum yields and the excited state’s lifetime unpredictably depend on the nature of the metal. Thus, typical f-elements, such as Eu^3+^, Tb^3+^, Dy^3+^, and Sm^3+^, as well as some IR-emitting ions, luminesce mainly due to the antenna sensitization mechanism [37]. For rhenium and platinum metal complexes, the metal orbitals play an important part in the electronic transitions that cause luminescence [4,5]. At the same time, cations with an electronic configuration, such as that of noble gases, i.e., Al^3+^, Ga^3+^, In^3+^, Sc^3+^, and La^3+^ (as well as the Lu^3+^ ion, which has the electronic configuration [Xe]4f^14^), influence the ligand luminescence not through the internal electronic transitions or the participation of valence orbitals, but only by changing the ligand space structure and by influencing the charge field on it. They are usually characterized by fluorescence with low lifetimes, and the emission color, depending on the ligand, lies in the violet-blue region [38]. The Gd^3+^ ion may exhibit its own 4f-f electronic transitions, but due to the high resonance level energy (>30,000 cm^−1^) [37] and paramagnetism-related effects, gadolinium complexes are characterized by phosphorescence in the blue-green spectral region [39]. 

To date, among the thirteen group metals (Al, Ga, and In), only one Ga^3+^ acylpyrazolonate complex has been described [40]. While aluminum compounds (such as 8-oxyquinolinate [41]) have played a crucial role in the history of OLED technology, the lack of research on group 13 acylpyrazolonates poses a certain challenge.

Corresponding lanthanide derivatives have been widely investigated, but in most of cases the attention has been paid on the luminescence properties of emitting f-elements, mainly terbium and europium [27,28]. 

Here, we report two series of coordination compounds obtained from the reaction of the trivalent metals, i.e., the 13 group metals (Al^3+^, Ga^3+^, and In^3+^), 3 group metals (Sc^3+^, La^3+^, Gd^3+^, and Lu^3+^), and the proligand (4-(cyclohexanecarbonyl)-5-methyl-2-phenyl-2,4-dihydro-3H-pyrazol-3-one **HQ^CH^**, with the aim of qualitatively and quantitatively correlating the photophysical ligand parameters to the central ion choice. We select this ligand since it allows the synthesis of compounds containing ions of various radii from Al^3+^ to Lu^3+^. Earlier, we showed that Q^CH^ lanthanide complexes exhibit excellent luminescent properties [42,43], and that the cyclohexyl substituent prevents the formation of molecular aggregates due to the molecular volume [44]. 

In order to disclose the influence of different metal ions on the ligand’s electronic structure, we also investigated in detail the photophysical properties of all the complexes by exploring the absorption, excitation, emission spectra, quantum yields of luminescence, and the lifetime of the excited states. All of the complexes reported here exhibit strong emissions in the blue-green region of the spectra, which makes them promising for use as blue emitting layer components in WOLEDs.

## 2. Results

### 2.1. Synthesis of Complexes

**Scheme 1:**
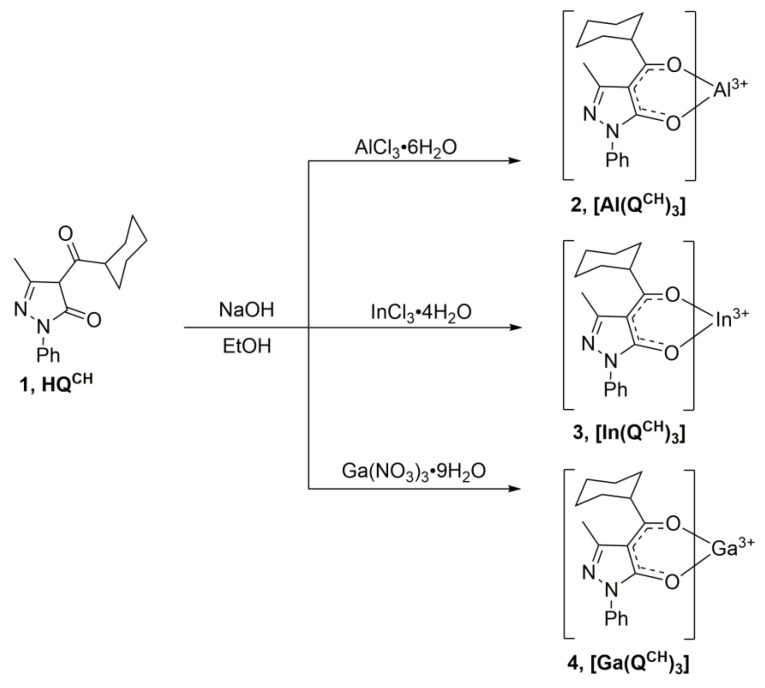
Preparation of Al^3+^, Ga^3+^, and In^3+^ complexes (**2**–**4**).

The complexes [M(Q^CH^)_3_] **2**–**4** (M = Al, Ga, and In) of all the three elements can be readily prepared in high yields in aqueous EtOH media, using hydrated salts as the metal precursors and NaOH as the base (Scheme 1): 

All three complexes were obtained in an anhydrous form and can be purified by recrystallization from hot EtOH. Gallium (III) nitrate was used as a source of Ga^3+^ ion as it is the most soluble salt. The nature of the anion did not affect the yield of the complexes.

It is worth noting that the Al^3+^, Ga^3+^, and In^3+^ complexes containing acylpyrazolonates have been scarcely studied to date [26]. Several aluminum complexes have been previously obtained from the interaction of Al(i-PrO)_3_ or anhydrous AlCl_3_ with three different 4-acylpyrazolones-5, bearing 4-acetyl, 4-benzyl, or 4-propionyl fragments in benzene [45]. Analogous In^3+^ complexes were prepared using In(i-PrO)_3_ as the starting material with a method also employed for the preparation of Al^3+^ ion complexes [46]. Al^3+^ and In^3+^ ion derivatives of 1-phenyl-3-methyl-4-trifluoroacetyl-pyrazolonate [47] and a binuclear In^3+^ complex with the multitopic 1,10-bis(1-phenyl-3-methyl-5-hydroxy-4-pyrazolyl)-1,10-decanedionate were synthetized and investigated in the extraction of indium and aluminum from the solutions [48]. Only Ga(1-phenyl-3-methyl-4-benzoyl-pyrazolonate) was reported in the literature without a detailed description of its preparation [49].

**Scheme 2:**
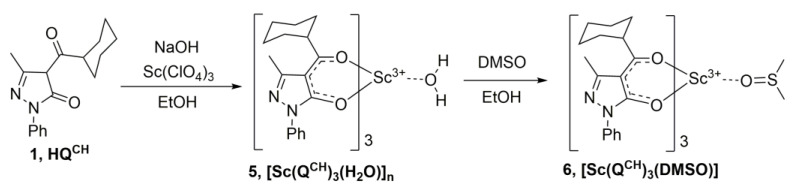
Preparation of Sc^3+^ complexes.

The interaction of **HQ^CH^** (**1**) with Sc(ClO_4_)_3_ and NaOH in the EtOH-H_2_O mixture led to the formation of [Sc(Q^CH^)_3_(H_2_O)]_n_ (**5**), as it was confirmed by an EA and FTIR data. This complex is insoluble in most solvents, including alcohols, which may testify to its polymeric structure with bridge water molecules. Previously, we have shown that for Sc^3+^ diketonates with heterocyclic ligands, a coordination number (CN) equal to seven is preferable [15]. This complex is readily soluble in coordinating solvents, such as DMSO, upon gentle heating. A ligand exchange occurs due to dissolution with the formation of a monomeric species (Scheme 2). 

Notably, upon the slow diffusion of EtOH vapor into a saturated DMSO solution of [Sc(Q^CH^)_3_(H_2_O)]_n_ (**5**) fine crystals of [Sc(Q^CH^)_3_(DMSO)] (**6**), a complex formed as a sole product due to the higher coordination ability of DMSO over EtOH. The choice of Sc(ClO_4_)_3_ is not critical for the synthesis; other soluble salts, such as chlorides or nitrates, can be used. However, it is more convenient to dissolve Sc_2_O_3_ in non-volatile HClO_4_ rather than in HCl or HNO_3_. 

**Scheme 3:**
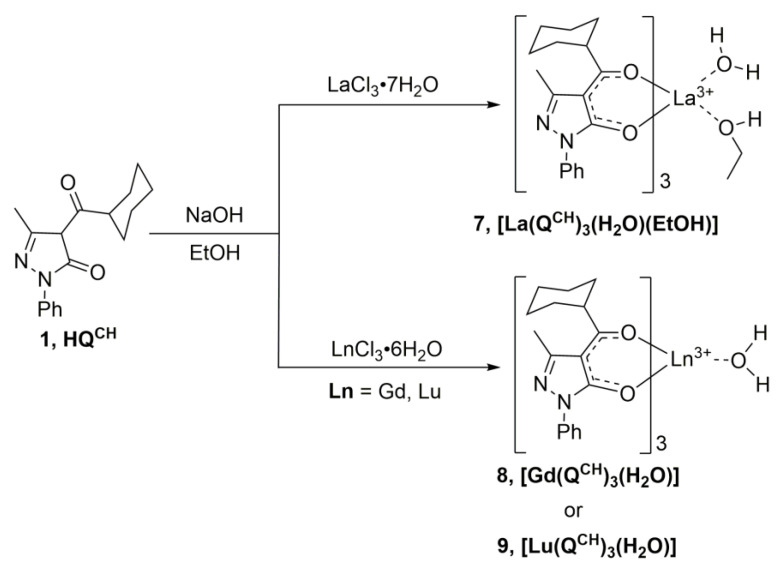
Preparation of La^3+^, Gd^3+^, and Lu^3+^ complexes (**7**–**9**).

For scandium (III), only one complex with 1-phenyl-3-methyl-4-benzoyl-pyrazolone-5 (**HL_1_**) was reported [50]. It was obtained by an interaction between the free ligand, hydrated Sc(NO_3_)_3_ in MeOH without a base, and identified as [Sc(L_1_)_3_]·H_2_O on the basis of an elemental analysis (EA) and FTIR data. Upon crystallization from hot MeOH, this amorphous complex transformed into anhydrous crystalline [Sc(L_1_)_3_], but no crystal structure data were provided. Complexes of La^3+^ and Lu^3+^ ions were obtained by a modified method, which has been described previously in the literature for other lanthanides [42,51,52] (Scheme 3):

Since the ionic radius of La^3+^ is bigger than that of Lu^3+^ due to lanthanide contraction, La^3+^ demonstrates a higher CN (8) and adopts two additional ligands (EtOH and water molecules) alongside three bulky diketonate anions. For the Gd^3+^ ion and especially Lu^3+^, the ion coordination number is 7, and only one additional water molecule can be inserted in the inner sphere of the complex together with three anions of Q^CH^ ligands. From hot EtOH complex [La(Q^CH^)_3_(H_2_O)(EtOH)] (**7**) crystallized as a solvate with one molecule of EtOH, but it can be fully desolvated by heating at 45 °C at a diminished pressure.

### 2.2. Single-Crystal X-ray Structures

#### 2.2.1. Complexes with p-Metals

Pale brown crystals of complexes [Al(Q^CH^)_3_] (**2**), [In(Q^CH^)_3_] (**3**) and[Ga(Q^CH^)_3_] (**4**), which are suitable for single-crystal X-ray diffraction, were obtained by the slow evaporation of solutions in MeOH or EtOH at room temperature. The selected crystal data and refinement parameters for complexes [Al(Q^CH^)_3_], [Ga(Q^CH^)_3_], and [In(Q^CH^)_3_] are listed in Table 1. The isostructurality of single-crystals to polycrystalline bulk samples was confirmed by the powder X-ray diffraction method (PXRD) (Figures S1–S3).

**Figure 1:**
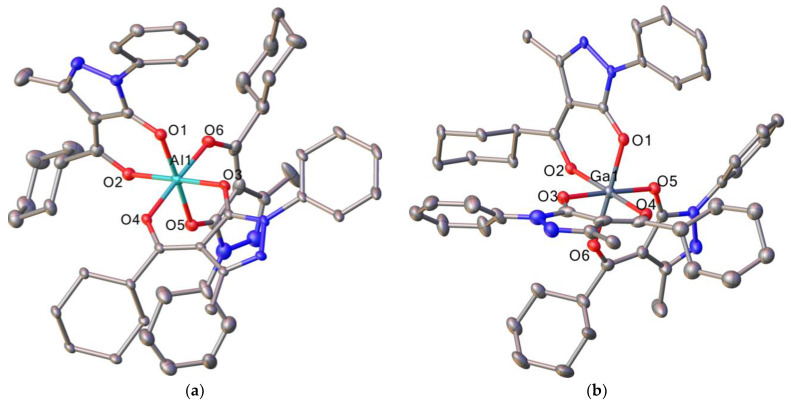
View of the asymmetric unit of [Al(Q^CH^)_3_] (**a**) and [Ga(Q^CH^)_3_] (**b**). Hydrogen atoms are not shown for clarity. Thermal ellipsoids are illustrated at 30% probability. Central metal atoms and oxygen atoms of ligands are labeled. Atoms are marked by colors as follows: N—blue, O—red, Al—turquoise, Ga—silver, C—gray.

**Figure 2:**
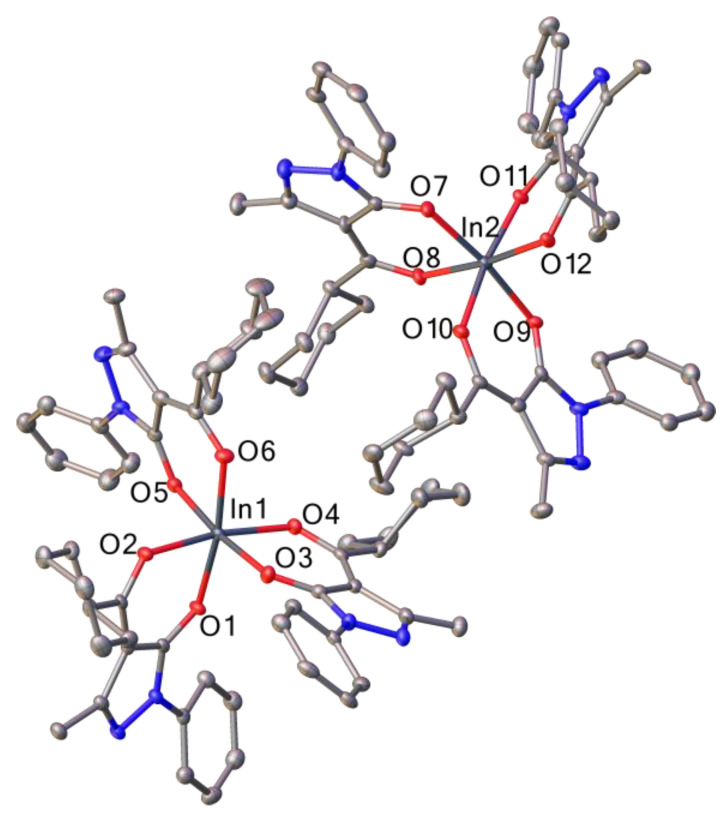
View of the asymmetric unit of [In(Q^CH^)_3_]. Hydrogen atoms are not shown for clarity. Thermal ellipsoids are illustrated at 50% probability. Central metal atoms and oxygen atoms of ligands are labeled. Atoms are marked by colors as follows: N—blue, O—red, In—silver, C—gray.

All three structures are mononuclear complexes, where the metal ion is coordinated by three diketonate ligands (Figure 1 and Figure 2), and according to the CCDC analysis, these structures present the first example of such complexes of Al^3+^, Ga^3+^, and In^3+^ ions with β-diketones. It must be noted that the asymmetric unit of the [In(Q^CH^)_3_] (**3**) crystal structure contains two molecules of the complex (Figure 2). Each central ion (Al^3+^, Ga^3+^, and In^3+^) bonds with two oxygen atoms of each ligand, leading to the octahedral coordination polyhedron {MO_6_} and neutral charge of the complexes. As for the {InO_6_} polyhedron, moderate distortion of the angle between two vertices can be observed, leading the O1-In1-O6 and O7-In2-O9 angles to be 171.2 and 173.2° instead of 180° for the ideal octahedron. The elongation of M-O bonds (Table S1) is observed for complexes [Al(Q^CH^)_3_], [Ga(Q^CH^)_3_], and [In(Q^CH^)_3_], which is attributed to the increase in the ionic radius of the central metal ion. The analysis of crystal packing revealed no presence of any hydrogen bonds, but the presence of rather weak C-H…π was observed.

#### 2.2.2. Complexes with Rare Earth Elements

Colorless crystals of complexes [Sc(Q^CH^)_3_(DMSO)] (**6**), [La(Q^CH^)_3_(H_2_O)(EtOH)]∙(EtOH) (**7**), [Gd(Q^CH^)_3_(H_2_O)] (**8**), and [Lu(Q^CH^)_3_(DMSO)] (**9**), which are suitable for single-crystal X-ray diffraction, were obtained by the slow evaporation of solutions in EtOH for La^3+^ and Gd^3+^ ion complexes or by the slow diffusion of EtOH vapors into a saturated DMSO solutions of complexes for Lu^3+^ and Sc^3+^ ion complexes at room temperature. The selected crystal data and refinement parameters for complexes [Sc(Q^CH^)_3_(DMSO)], [La(Q^CH^)_3_(H_2_O)(EtOH)]∙(EtOH), [Gd(Q^CH^)_3_(H_2_O)], and [Lu(Q^CH^)_3_(DMSO)] are listed in Table 2. The isomorphism of the crystal structures of the studied single crystals [La(Q^CH^)_3_(H_2_O)(EtOH)]∙(EtOH) and [Gd(Q^CH^)_3_(H_2_O)] and the corresponding bulk material was confirmed by PXRD (Figures S4 and S6).

**Figure 3:**
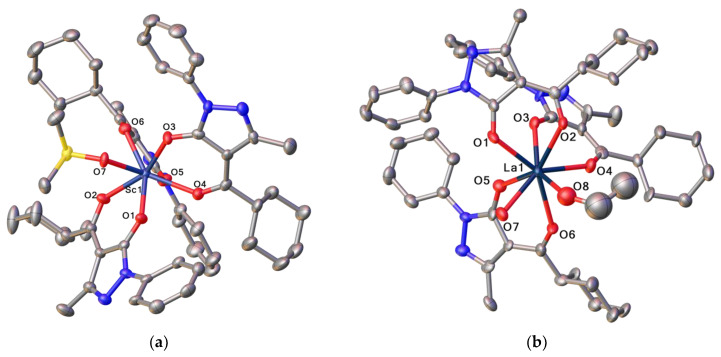
View of the asymmetric unit of [Sc(Q^CH^)_3_(DMSO)] (**a**) and [La(Q^CH^)_3_(H_2_O)(EtOH)]∙(EtOH) (**b**). Hydrogen atoms and solvated EtOH molecule in (**b**) are not shown for clarity. Thermal ellipsoids are illustrated at 50% probability. Central metal atoms and oxygen atoms of ligands are labeled. Atoms are marked by colors as follows: N—blue, O—red, Sc—silver-blue, La—dark blue, S—yellow, C—gray.

**Figure 4:**
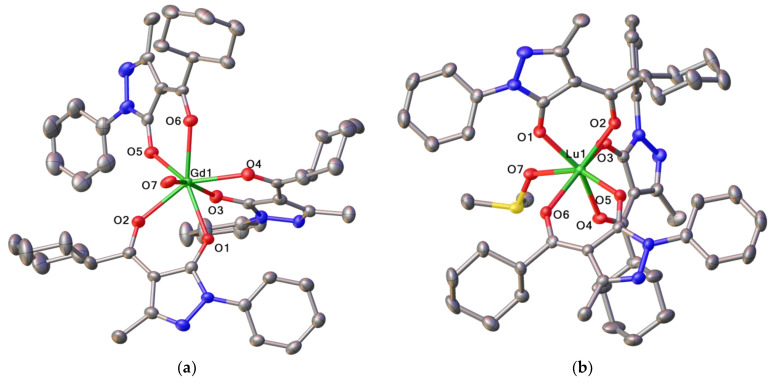
View of the asymmetric unit of [Gd(Q^CH^)_3_(H_2_O)] (**a**) and [Lu(Q^CH^)_3_(DMSO) (**b**). Hydrogen atoms are not shown for clarity. Thermal ellipsoids are illustrated at 50% probability. Central metal atoms and oxygen atoms of ligands are labeled. Atoms are marked by colors as follows: N—blue, O—red, Gd or Lu—green, S—yellow, C—gray.

All four structures are mononuclear complexes, where the metal ion is coordinated by oxygen atoms of three diketonate ligands (Figure 3 and Figure 4) and by oxygen atoms of a number of solvate molecules (one DMSO for [Sc(Q^CH^)_3_(DMSO)] and [Lu(Q^CH^)_3_(DMSO)], one water and one EtOH for [La(Q^CH^)_3_(H_2_O)(EtOH)]∙(EtOH), and one water for [Gd(Q^CH^)_3_(H_2_O)]). It should be noted that [Sc(Q^CH^)_3_(DMSO)] and [Lu(Q^CH^)_3_(DMSO)] complexes are isostructural. It should also be noted that, according to the PXRD data (Figure S5), the [Sc(Q^CH^)_3_(H_2_O)] and [Lu(Q^CH^)_3_(H_2_O)] complexes also turned out to be isostructural; however, due to the depressingly low solubility levels in most of the weak or moderate coordinating solvents, it was not possible to grow the single crystals of these compounds.

At the same time, the slow diffusion of EtOH vapors into the solutions of hydrated complexes in DMSO led to the other crystal structures, namely [Sc(Q^CH^)_3_(DMSO)] (**6**) or [Lu(Q^CH^)_3_(DMSO)] (**9**), having one coordinated DMSO molecule instead of a water molecule. This alteration does not affect the coordination polyhedron {MO7}, which is a capped trigonal prism in all cases, but rather leads to a slight change in the relative arrangement of the ligands around the metal ion (Figure S7).

As for the [La(Q^CH^)_3_(H_2_O)(EtOH)]∙(EtOH) (**7**) complex, a relatively larger ionic radius of La^3+^ compared to Sc^3+^, Gd^3+^, and Lu^3+^ ions causes the formation of an octa-coordinated complex that includes two solvent molecules in the inner coordination sphere, leading to the {MO_8_} La^3+^ polyhedron, which is best described as a square antiprism. 

The analysis of Ln^3+^-O bond lengths in the corresponding La^3+^, Gd^3+^, and Lu^3+^ ion complexes (Table S2) allows one to observe the influence of lanthanide contraction, which results not only in the shortening of bond lengths, but also in changes in the coordination number from eight for [La(Q^CH^)_3_(H_2_O)(EtOH)]∙(EtOH) to seven for [Gd(Q^CH^)_3_(H_2_O)] and [Lu(Q^CH^)_3_(DMSO)]. It is also worth noting that the hepta-coordinated lanthanide ion is observed in Eu^3+^ [29] and Dy^3+^ [42] complexes that are isostructural to [Gd(Q^CH^)_3_(H_2_O)]. 

The analysis of the crystal packing of isostructural [Sc(Q^CH^)_3_(DMSO)] and [Lu(Q^CH^)_3_(DMSO)] complexes did not reveal any strong intermolecular hydrogen bonds besides rather weak CH…π interactions. On the contrary, the crystal packing of [Gd(Q^CH^)_3_(H_2_O)] additionally contains a number of O-H…N hydrogen bonds (the O7…N2 distance is 2.776 Å; the O7…N4 distance is 2.703 Å, see Figure S8). As for [La(Q^CH^)_3_(H_2_O)(EtOH)]∙(EtOH), a solvated EtOH molecule assists in the formation of a large amount of O-H…O and O-H…N intermolecular interactions (the O7…N6 distance is 2.791 Å; the O8…O9 distance is 2.934 Å; the O9…N2 distance is 2.720 Å, see Figure S9)

### 2.3. Spectroscopic Studies

**Figure 5:**
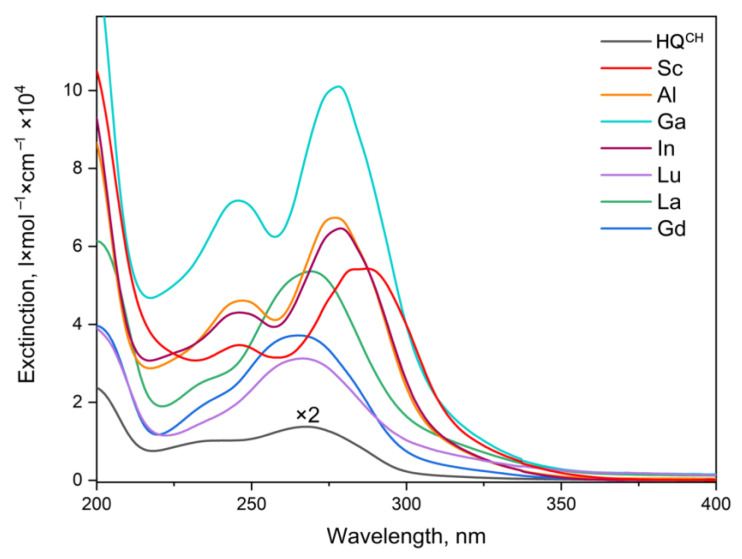
UV–Vis spectra for the compounds in acetonitrile (MeCN).

Optical absorption spectra were obtained for all the complexes and **HQ^CH^ (1)**. As can be seen in Figure 5, the absorption spectra exhibit two pronounced absorption bands located in the UV region of spectra. The bands correspond to ligand absorption and we observed no ion absorption for any of the complexes. The spectra recorded for the complexes [La(Q^CH^)_3_(H_2_O)(EtOH)], [Lu(Q^CH^)_3_(H_2_O)], and [Gd(Q^CH^)_3_(H_2_O)], designated further as **La**, **Lu**, and **Gd**, respectively, qualitatively resemble the spectrum of **HQ^CH^**. However, the molar extinction (ε) increases from 6 × 10^3^ for **HQ^CH^** to 3.2–5.5 × 10^4^ $L\times mol^{-1}\times cm^{-1}$ for complexes. Moreover,ε increases monotonically from 2.2 × 10^4^ to 5.5 × 10^4^ $L\times mol^{-1}\times cm^{-1}$ with the replacement of the central ion by one with a higher atomic number. On the contrary, the 13 group metal and Sc^3+^ ions affect the optical absorption of the complexes [Sc(Q^CH^)_3_(H_2_O)] and [M(Q^CH^)_3_], M = Al, In, and Ga (designated as **Sc**, **Al**, **In**, and **Ga**, respectively), by two factors. Firstly, they multiply ligand extinction up to 10 times. Secondly, these complexes have the red-shift of absorption bands in comparison with the complexes of lanthanide ions and **HQ^CH^**. The maximum red-shift of an absorption band is observed for the **Sc** complex. Notably, the absorption bands are better resolved in the spectra recorded for the **Al**, **Sc**, **In**, and **Ga** complexes than that in the spectra recorded for **HQ^CH^** and lanthanide ion complexes. This is caused by the redistribution of the oscillator strengths corresponding to these bands [15].

**Figure 6:**
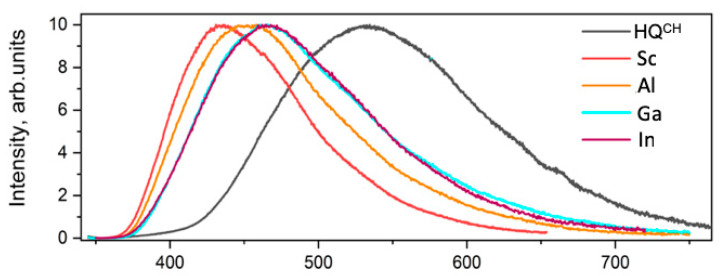

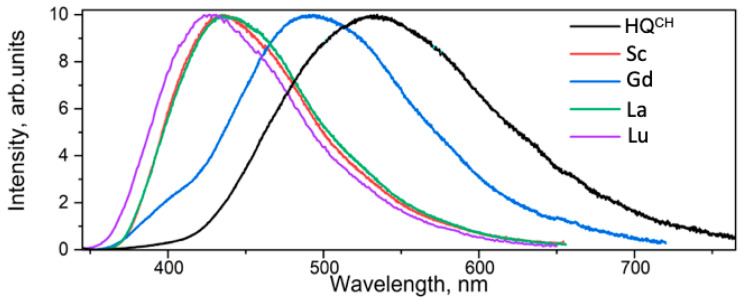
Photoluminescence spectra for the compounds in solid state under CW excitation at 340 nm.

Photoluminescence (PL) spectra, measured under excitations at 340 nm wavelengths, are shown on Figure 6. The spectrum of **H → Q^CH^** reveals a wide spectral band (FWHM = 92 nm) with the emission maximum at 530 nm and with a wide shoulder at longer wavelengths up to 800 nm. Additionally, the additional fluorescence band appears within the region of 380–420 nm. We observed significant influences of the central ion on the PL spectrum. Actually, the emission maximum (λ_em_) shifts to the blue region of the optical spectrum from 530 nm for the **HQ^CH^** ligand to 490 nm for **Gd**, 464 nm for **In** and **Ga**, and 458 nm for **Al**. Finally, the maximum blue-shift of the PL maximum is for **Sc**, **La**, and **Lu**, measuring 430, 436, and 428 nm, respectively. Surprisingly, the spectrum taken for **Gd** exhibits a wide spectral band (FWHM = 104 nm) and the maximum is centered at 490 nm. There is a low intensity spectral band located within 380–420 nm, which matches with the PL maxima of the **Sc**, **La**, and **Lu** complexes. We suppose that the redistribution of the emission intensity is determined by the presence of dual emission: ligand phosphorescence located at longer wavelengths and ligand fluorescence at 425 nm.

**Figure 7:**
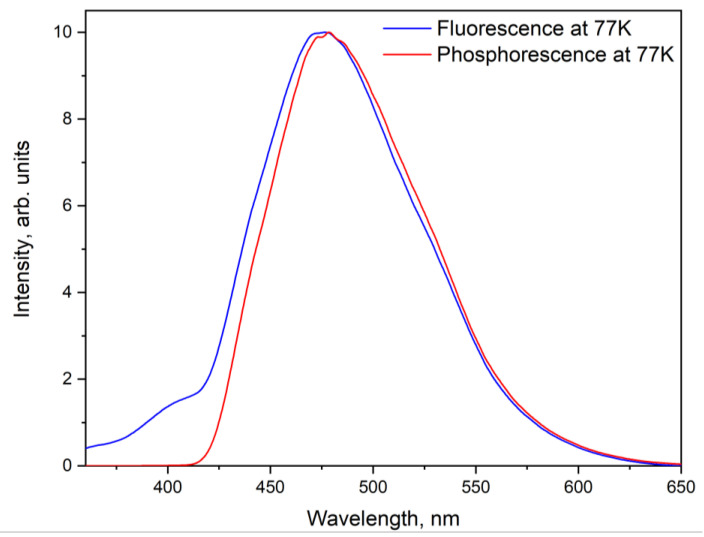
Photoluminescence spectra of [Gd(Q^CH^)_3_(H_2_O)] (**8**) at temperature 77 K.

To check this hypothesis, the fluorescence and phosphorescence spectra at 77 K were measured (see Figure 7). It is clearly noticeable that the spectral band in the short-wave region of the phosphorescence spectrum disappears, while in the spectrum without delay (fluorescence spectrum), the band is still observed, which unequivocally confirms the fluorescent nature of this spectral band.

A comparison between the spectra recorded for the complexes of lanthanide ions and those for the complexes of the 13 group metals implies that the valence electrons caused the shift of the emission maximum toward 458 nm for **Al** and 464 nm for **In**.

**Figure 8:**
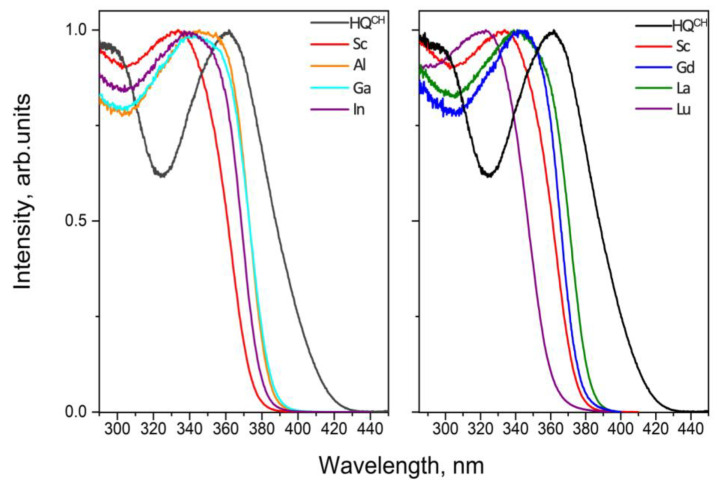
PL excitation spectra for compounds at solid state with registration wavelengths at PL maxima.

Photoluminescence excitation (PL) spectra were obtained for all the compounds and **HQ^CH^** with the registration wavelength located at the emission maxima, respectively. All the spectra are qualitatively similar, revealing a broad excitation band at 320–400 nm. An excitation maximum of 361 nm was estimated for the free ligand (**HQ^CH^**) under emission registration at 530 nm, whereas the spectra for complexes revealed a blue-shifted excitation band with the maxima located in a neighborhood of 340 nm, except for the **Lu** complex (see Figure 8 and Table 3).

To gain insight into the electronic excitation relaxation processes in the investigated compounds, luminescence decays were recorded. All the experiments were conducted at room temperature. As seen from Table 3 (see Figure S10), the formation of trivalent ion complexes increases the observable luminescence lifetime (τ) in comparison with **HQ^CH^**. The luminescence decays recorded for the *p*-metal ion complexes **Al**, **Ga**, and **In** reveal single exponential behavior with characteristic lifetimes of τ = 9.9, 6.3, and 4.2 ns, respectively. Therefore, the replacement of the central Al^3+^ ion with the heavier one (Ga^3+^ and In^3+^) leads to a decrease in the observed lifetimes of up to two times. On the contrary, the decays obtained for rare earth ion complexes have more complicated behaviors. The multiexponential law can fit the recorded kinetic traces:

(1) $$I_{th}\left(t\right)=\sum_{i=1}^{n}A_{i}e^{-\frac{t}{\tau_{i}}}$$

where τ_i_ and A_i_ are decay times and amplitudes, respectively. The measured luminescence decay is determined by the following equation:

(2) $$I_{e\mathrm{x;}p}\left(t\right)=\int_{0}^{\infty }I_{irf}\left(t^{\prime }\right)I_{th}\left(t-t^{\prime }\right)dt^{\prime }$$

where $I_{irf}\left(t^{\prime }\right)$ is the instrument response function (IRF), which can be described as a double Gaussian shape with the characteristic lifetime of τ_irf_ = 0.5 ns.

Specifically, the decays for the complexes of the La^3+^, Lu^3+^, and Sc^3+^ ions fit with the bi-exponential function. The presence of two relaxation components in the fluorescence decays of the Sc, La, and Lu complexes can be attributed to the distinct emitting sites that are responsible for luminescence [15]. Their characteristic lifetimes are listed in Table 3. Unexpectedly, the luminescence of the **Gd** compound has a significantly longer decay with characteristic lifetimes of τ_1_ = 22 μs and τ_2_ = 36 μs (see Figure S11). Therefore, the relatively long lifetime proves the phosphorescence nature of long wavelength bands in the PL spectrum for **Gd**. We also measured the luminescence decay for the **Gd** compound at a cryogenic temperature of 77 K. Cooling leads to the suppression of all the rotational–vibrational processes with consequentially higher values of characteristic lifetimes of τ^77^_1_ = 220 μs and τ^77^_2_ = 536 μs.

## 3. Discussion

The energies of the first excited singlet and triplet states were estimated by generally recognized methods [25,53]. Due to the energy reorganization in absorption and emission processes for the non-adiabatic approximation, energies of the S_0_ → S_1_ and T_1_ → S_0_ transitions can be determined as the low-energy edges of the absorption spectrum and high-energy edges of the phosphorescence spectrum with the use of the tangent method [25]. To suppress the rotational–vibrational processes during phosphorescence measurements, the complexes were cooled down to 77 K. To remove the fast fluorescence contribution, a 200 μs delay was employed. The estimated energies of the T_1_ state are listed in Table 3. We obtained the close values for all the complexes, which lie in the range of 23,500–23,700 cm^−1^, except for Sc. An energy of 22,715 cm^−1^ was obtained for Sc. Therefore, the central ion leads to a T_1_ state energy increase from 22,100 cm^−1^ for **HQ^CH^** to 22,715 cm^−1^ for **Sc** and approximately 23,600 cm^−1^ for all other complexes.

The first excited singlet state energy (S_1_) increases for all the complexes in comparison with **HQ^CH^**, which has an S_1_ energy of 26,000 cm^−1^. The highest energy was obtained for Lu—28,000 cm^−1^; other complexes’ energies lay in the range of 27,000–27,700 cm^−1^. Thereby, we did not observe significant changes in the energy gap (ΔE_ST_) between the S_1_ and T_1_ states due to the influence of ion substitution (see Table 3).

It should be noted that the ΔE_ST_ values of the **La** and **Gd** complexes are sufficiently close to the ligand’s values. As ΔE_ST_ values of **La** and **Gd** and the ligand are close, and the **La** and **Gd** absorption spectra qualitatively resemble the ligand’s one, we consider that, specifically, La^3+^ and Gd^3+^ do not distort the potential energy surfaces of the S_1_ and T_1_ states.

Figure 7 demonstrates the fluorescence and phosphorescence spectra obtained for the **Gd** complex. The emission spectrum, recorded at 77 K, reveals two emission bands located at 380–420 nm and at 420–650 nm. The band located in the region 380–420 nm vanishes in the phosphorescence spectrum, proving the presence of two radiative relaxation processes with different emission states for the **Gd** complex. Namely, fluorescence appears within 380–420 nm and phosphorescence is observed on a long-wavelength spectral range. It is interesting that, according to the literature, room temperature phosphorescence is quite rare to see [7,54,55]. 

Therefore, there are two possible explanations for this phenomenon. First, the intersystem crossing process (ISC) of the **Gd** complex has a higher rate compared with the other complexes. The second explanation is that it has a much lower rate of non-radiative processes due to the different molecule structures and different symmetry groups in particular.

Two observed lifetimes can be assigned to radiative relaxation from two local minimums on the T_1_ state’s potential energy surface (PES). Since the long time component’s lifetime is much higher under cooling than the short time component’s lifetime, we conclude that the longest time component (τ_2_) is associated with radiative relaxation from the deepest minimum of T_1_ PES. For all the investigated complexes, we measured the PL quantum yield values Φ under optical excitations at 340 nm, providing excitations in the maximum of the luminescence excitation spectra (see Figure 4). As follows from Table 3, the formation of complexes leads to strong increases in the PL quantum yield by up to 39 times. In particular, the maximum Φ value was recorded for **La**. In this compound ligand, the fluorescence Φ was enhanced from 0.5% to 19.5%. In the 13 group metal complexes, the replacement of the central ion with a heavier one led to a decrease in the quantum yield value from 16.9% for **Al** to 6.6% for **Ga** and 3.3% for **In**. We see the same dependence for the **La** (19.5%) and **Lu** (6.5%) complexes. The probabilities of radiative (k_rad_) and non-radiative (k_nrad_) processes were evaluated using the following formulas [53]:

(3) $$k_{rad}=\frac{\Phi }{\tau_{obs}}$$

(4) $$\tau_{obs}=\frac{1}{k_{rad}+k_{nrad}}$$

(5) $$k_{nrad}=\frac{1}{\tau_{obs}}-k_{rad}$$

As can be seen from Table 3 and Table 4, the rate of the radiative process monotonically reduces from 1.7 × 10^7^ to 0.7 × 10^7^ s^−1^, and the quantum yield decreases from 16.9 to 3.0 under the replacement of the central Al^3+^ ion on Ga^3+^ and In^3+^. Notably, the fluorescence quantum yield value recorded for the **Gd** complex is only 1.8%, since the radiative relaxation of S_1_ is a less pronounced pathway than the intersystem crossing process (ISC) followed by phosphorescence. As we noted a significant increase in the observed luminescence lifetime under cooling up to 77 K, a huge enhancement of the quantum yield was expected. While the number of emitted photons equals the integrated intensity of the emission spectrum, the luminescence quantum yield at a temperature of 77 K (Φ_77_) can be calculated by the following formula:

(6) $$\Phi_{77}=\frac{I_{77}}{I_{300}}\Phi_{300}$$

where I_77_ integrated luminescence intensity at 77 K, I_300_ integrated luminescence intensity at 300 K, and Φ_300_ quantum yield at 300 K. According to this procedure, we achieved the quantum yield value of 45.8% for the **Gd** complex at a temperature of 77 K. 

To estimate the energy transfer process from a singlet state to a triplet manifold, the intersystem crossing rates (k_isc_) were calculated using emission lifetimes and fluorescence quantum yields, both at 300 K and 77 K. Since only the **Al**, **In**, **Ga**, and **Gd** complexes and HQ^CH^ demonstrate the single exponential fluorescence behavior, calculations were only performed for these compounds. The intersystem crossing rate can be evaluated as follows [25]:

(7) $$k_{isc}=\frac{1-\Phi }{\tau_{obs}}.$$

After comparing the ISC rates obtained at 300 K and 77 K, we conclude that the rates for the **Al**, **In**, and **Ga** complexes are lower at 77 K. This is caused by significant vibrational relaxation at room temperature. Taking into account the fact that the probability of non-radiative vibrational relaxation processes is negligibly low at 77 K, we assume that the applied method is more beneficial for calculations at 77 K. The rates calculated at 77 K increase with an increase in the atomic number of the 13 group ions. The k_isc_ rate of the **HQ^CH^** rate remained unchanged with the decrease in the temperature (13.4 × 10^7^ s^−1^ and 13.3 × 10^7^ s^−1^ at 300 K and 77 K, respectively), suggesting that phosphorescence is predominant in the relaxation channel (see Figure 6). On the contrary, the **Gd** complex rate slightly increases from 12.5 × 10^7^ s^−1^ (300 K) to 13.8 × 10^7^ s^−1^ (77 K) due to the paramagnetic properties of the Gd^3+^ ion (See Table 4). Notably, since the **Gd** compound has a rigid geometry, vibrational relaxation is reduced in comparison with **HQ^CH^**. Therefore, phosphorescence in the **Gd** complex is more effective relaxation pathway (see Table 3).

## 4. Materials and Methods

Common reagents were purchased form Aldrich (St. Louis, MO, USA) and were used without further purification. Ligand-1-phenyl-3-methyl-4-cyclohexylcarbonyl-pyrazol-5-one (**HQ^CH^, 1**) was synthesized according to previously published procedure [42]. Rare earth compounds of high purity (99.99–99.999%) were purchased from Lanhit (Moscow, Russia).

Stock 1 M Sc(ClO_4_)_3_ solution was prepared as follows: after being freshly calcinated at 600 °C, Sc_2_O_3_ (6.896 g, 50 mmol, 99.999%) was dissolved upon heating in a quartz flask in mixture of 26 mL of perchloric acid (70%, 99.999% trace metals basis, Aldrich) and 20 mL of deionized water. Excess water was slowly evaporated at 90 °C; the residue was quantitatively transferred to a 100 mL volumetric flask and brought to volume by deionized water. Solution was stored in a polypropylene bottle. 

Elemental analysis was performed by Elemental Vario MicroCube CHNO(S) analyzer (Elementar Analysensysteme, Langenselbold, Germany). The metal content was determined by complexometric titration with a Trilon B (disodium salt of ethylenediaminetetraacetic acid) solution in the presence of Xylenol Orange as an indicator (for scandium, lanthanum, and lutetium) or by ICP-MS analysis (for aluminum, gallium, and indium). Before the analysis, the complexes were decomposed by heating with concentrated HNO_3_. ICP-MS was performed using an inductively coupled plasma mass spectrometer ELAN mod. 9000, DRC II, DRC-e (PerkinElmer, Waltham, MA, USA). FTIR spectra were recorded in KBr pellets on Perkin Elmer Spectrum One instrument (PerkinElmer, Waltham, MA, USA). 

Single-crystal X-ray diffraction analysis of [Al(Q^CH^)_3_], [Ga(Q^CH^)_3_], [In(Q^CH^)_3_], [Sc(Q^CH^)_3_(DMSO)], [La(Q^CH^)_3_(H_2_O)(EtOH)]∙(EtOH), [Gd(Q^CH^)_3_(H_2_O)], and [Lu(Q^CH^)_3_(DMSO)] was carried out on a Bruker D8 Quest (Bruker, Billerica, MA, USA) diffractometer (MoKα radiation, ω and φ-scan mode). The structures were solved with direct methods and refined by least-squares method in the full-matrix anisotropic approximation on F^2^. High reported values of R_1_-factors for [Al(Q^CH^)_3_] and [Ga(Q^CH^)_3_] were due to their weak scattering of X-ray caused by disorders of cyclohexyl substituents. All hydrogen atoms were located in calculated positions and refined within riding model. All calculations were performed using the SHELXTL [56,57] and Olex2 [58] software packages. Atomic coordinates, bond lengths, angles, and thermal parameters have been deposited at the Cambridge Crystallographic Data Centre with deposition numbers—CCDC 2208569–2208571, 2215463, 2215494, 2215723, and 2215486, which are all available, free of charge at www.ccdc.cam.ac.uk (accessed on 3 April 2023).

Powder X-ray diffraction (PXRD) patterns were measured on D/MAX 2500 (Rigaku Corporation, Tokyo, Japan) diffractometer in the reflection mode with CuKα_1_ radiation (λ = 1.54056 Å) and curved graphite [002] monochromator placed in the reflected beam.

Optical absorption spectra of Ln^3+^ compounds and HL dissolved in acetonitrile (HPLC SuperGradient, Panreac, Spain) were recorded using JASCO V-770 (Jasco, Tokyo, Japan) spectrophotometer operating within 200–2500 nm. Concentrations of the solutions were approximately 10^−5^ M/L. For solutions, the measurements were performed using quartz cells with a 1 cm pathlength. Photoluminescence spectra and luminescence excitation spectra were measured using Horiba Jobin-Yvon Fluorolog QM-75-22-C spectrofluorimeter using a 75 W xenon arc lamp (PowerArc, HORIBA, Kyoto, Japan). A Hamamatsu R13456 (Hamamatsu Photonics, Hamamatsu, Japan) cooled photomultiplier tube sensitive in UV–Vis-NIR region (200–950 nm) was used as the detector. Photoluminescence quantum yields were obtained for solid samples by absolute method with the use of same experimental setup, which were equipped with integration sphere G8 (GMP, Renens, Switzerland). Photoluminescence decays were measured by time-correlated single photon counting (TCSPC) method using the same spectrofluorimeter. The setup included DeltaLED (HORIBA, Kyoto, Japan) as a pulsed excitation source emitting at 340 nm with a repetition rate of 6.25 MHz and pulse duration FWHM of 0.6 ns. For all optical measurements, the corresponding instrument response functions were taken into account. The experiments were performed in air at atmospheric pressure. Degradation of the optical properties was not observed during the experiments.

IR spectra were registered in the range 4000–400 cm^−1^ in KBr pellets using a Perkin-Elmer system Spectrum One 100 FTIR (PerkinElmer, Waltham, MA, USA) spectrometer. IR spectra of all complexes are given in Figure S12.

Nuclear magnetic resonance (NMR) spectra were recorded in (CD_3_)_2_CO or DMSO-*d_6_* solutions at 298 K on a Bruker AC-300 (Bruker, Billerica, MA, USA) spectrometer operating at 300.13 MHz for ^1^H. TMS (δ = 0.00 ppm), which was used as a standard. 

**Synthesis of [Al(Q^CH^)_3_] (2), [In(Q^CH^)_3_] (3) and [Ga(Q^CH^)_3_] (4)**

Ligand **HQ^CH^** (**1**) (0.426 g, 1.5 mmol) was dissolved in 10 mL of EtOH (96%) at 45 °C and 0.5 mmol solution of corresponding hydrated chloride or nitrate (0.121 g of AlCl_3_∙6H_2_O, 0.209 g of Ga(NO_3_)_3_∙9H_2_O, or 0.147 g InCl_3_∙4H_2_O) in 2 mL of boiling EtOH, was added slowly with continuous magnetic stirring. The mixture was stirred for 5 min at 45 °C, and then 1.5 mL (1.5 mmol) of 1 M NaOH solution in EtOH was added dropwise. The resulting solution was stirred in a closed vial for additional 4 h at 45 °C, cooled to room temperature, and precipitate was separated. The precipitate was washed successively with 8 mL of 20% aqueous EtOH, 8 mL of deionized water, and 5 mL of hexane, and dried at 40 °C and 0.1 torr to a constant weight. 

**Tris-(4-(cyclohexylcarbonyl)-5-methyl-2-phenyl-2,4-dihydro-3*H*-pyrazol-3-onato) aluminum (III), [Al(Q^CH^)_3_] (2)**

Slightly pink powder: yield is 0.364 g (83%). Anal. calcd. for C_51_H_57_AlN_6_O_6_ (877.02): C, 69.84; H, 6.55; N, 9.58; Al, 3.08; found: C, 69.89; H, 6.61; N, 9.65; Al, 3.11%. IR: 3061 w; 2929 m ν_as_ CH (cyclohexyl); 2855 m ν_s_ CH (cyclohexyl); 2664 w; 2532 w; 2353 w; 1943 w; 1869 w; 1793 w; 1612 vs. ν(C=O); 1596 s ν(C=C, C=N); 1584 s; 1537 s; 1519 s; 1498 vs. ν(C=C, C=N); 1465 s; 1442 s; 1415 m; 1391 s; 1364 w; 1351 w; 1246 w; 1178 w; 1158 w; 1141 w; 1126 w; 1083 s; 1066 m; 1027 m; 1010 w; 1001 w; 985 m δ_r_ (C-H cyclohexyl); 924 w; 907 w; 894 w; 873 w; 845 w; 821 m; 792 w; 771 w; 758 m; 725 w; 690 m; 660 m; 642 w; 632 w; 615 vw; 549 w; 524 w; 510 w; 497 w; 448 vw; 415 w; 407 w. ^1^H-NMR (acetone-d_6_): 8.08–7.72 (m, 2H, o-C_6_H_5_); 7.38 (t, 1.08H, p-C_6_H_5_); 7.28–7.08 (m, 1.95H, m-C_6_H_5_); 3.08 (m, 1.01H, HC-C=O); 2.47 (m, 3.16H, CH_3_), 1.89–1.09 (m, 10.33H, cyclohexyl).

**Tris-(4-(cyclohexylcarbonyl)-5-methyl-2-phenyl-2,4-dihydro-3*H*-pyrazol-3-onato) indium (III), [In(Q^CH^)_3_](3)**

Slightly yellow powder: yield is 0.354 g (77%). Anal. calcd. for C_51_H_57_InN_6_O_6_ (964.85): C, 63.49; H, 5.95; N, 8.71; In, 11.90; found: C, 63.44; H, 6.12; N, 8.90; In, 12.08%. IR: 3047 m; 2931 m ν_as_ CH (cyclohexyl); 2855 m ν_s_ CH (cyclohexyl); 2665 w; 2354 w; 2250 w; 1944 w; 1885 w; 1794 w; 1737 w; 1603 s ν(C=O); 1592 s ν(C=C, C=N); 1574 vs; 1535 s; 1485 vs. ν(C=C, C=N); 1461 s; 1441 m; 1405 m; 1382 s; 1362 m; 1350 w; 1323 w; 1238 w; 1178 w; 1140 w; 1077 m; 1066 m; 1027 w; 1011 w; 1001 w; 982 m δ_r_(C-H cyclohexyl); 922 w; 908 w; 893 w; 870 w; 842 w; 815 w; 791 w; 767 w; 757 m; 711 w; 690 w; 668 vw; 656 w; 644 vw; 622 w; 614 w; 511 w; 503 w; 466 w; 446 vw; 419 vw; 412 vw; 404 vw. ^1^H-NMR (acetone-d_6_): 7.89–7.85 (m, 2H, o-C_6_H_5_); 7.37–7.32 (m, 2.00H, m-C_6_H_5_); 7.22–7.19 (m, 1.04H, p-C_6_H_5_); 3.12 (m, 1.01H, HC-C=O); 2.45 (m, 3.07H, CH_3_), 1.90–1.09 (m, 10.44H, cyclohexyl).

**Tris-(4-(cyclohexylcarbonyl)-5-methyl-2-phenyl-2,4-dihydro-3*H*-pyrazol-3-onato) gallium (III), [Ga(Q^CH^)_3_](4)**

Slightly yellow powder: yield is 0.361 g (78%). Anal. calcd. for C_51_H_57_GaN_6_O_6_ (919.76): C, 66.60; H, 6.25; N, 9.14; Ga, 7.58; found: C, 66.64; H, 6.19; N, 9.21; Ga, 7.69%. IR: 3062 s; 2929 m ν_as_ CH (cyclohexyl); 2855 w ν_s_ CH (cyclohexyl); 2663 w; 2525 w; 2353 w; 1942 w; 1868 w; 1792 vs; 1606 vs. ν(C=O); 1594 vs. ν(C=C, C=N); 1579 s; 1535 vs; 1495 s ν(C=C, C=N); 1463 s; 1437 m; 1411 s; 1386 m; 1363 m; 1351 m; 1325 w; 1242 w; 1177 w; 1140 w; 1124 s; 1079 m; 1066 m; 1027 m; 1010 m; 984 s δ_r_(C-H cyclohexyl); 924 w; 907 w; 894 w; 872 w; 843 m; 818 m; 791 w; 768 m; 758 s; 716 w; 690 m; 659 m; 643 w; 625 m; 614 w; 509 w; 476 w; 448 w. ^1^H-NMR (acetone-d_6_): 8.00–7.78 (m, 2H, o-C_6_H_5_); 7.39 (t, 1.04H, p-C_6_H_5_); 7.26–7.12 (m, 2.03H, m-C_6_H_5_); 3.09 (m, 1.02H, HC-C=O); 2.48 (m, 3.12H, CH_3_), 1.90–1.09 (m, 10.32H, cyclohexyl).

**Polymeric tris-(4-(cyclohexylcarbonyl)-5-methyl-2-phenyl-2,4-dihydro-3*H*- pyrazol-3-onato)(aqua) scandium (III), [Sc(Q^CH^)_3_(H_2_O)]_n_ (5)**

Ligand **HQ^CH^** (**1**) (0.426 g, 1.5 mmol) was dissolved in 10 mL of EtOH (96%) at 45 °C and 0.5 mL (0.5 mmol) of 1 M aqueous Sc(ClO_4_)_3_ solution was added, followed by slow addition of 1.5 mL (1.5 mmol) of 1 M ethanolic NaOH solution. Light gray precipitate formed immediately, and the resulting thick suspension was stirred at 45 °C for 5 h and cooled to room temperature. The precipitate was filtered off, washed successively with 10 mL of 50% aqueous EtOH, 20 mL of deionized water, and 10 mL of hexane, and dried at 40 °C and 0.1 torr to a constant weight. 

Light gray powder: yield is 0.424 g (93%). Anal. calcd. for C_51_H_59_N_6_O_7_Sc (913.01): C, 67.09; H, 6.51; N, 9.20; Sc, 4.92; found: C, 67.15; H, 6.58; N, 9.15; Sc, 5.04%. IR: 3059 m; 2930 s ν_as_ CH (cyclohexyl); 2849 m ν_s_ CH (cyclohexyl); 1937 w; 1864 w; 1655 m δ(H_2_O); 1616 vs. ν(C=O); 1595 s ν(C=C, C=N); 1582 s; 1533 s; 1501 s; 1486 s ν(C=C, C=N); 1464 m; 1451 s; 1440 s; 1402 m; 1382 m; 1350 m; 1326 w; 1269 w; 1239 w; 1210 w; 1178 w; 1130 w; 1114 w; 1082 m; 1030 w; 1011 w; 1000 w; 981 m δ_r_(C-H cyclohexyl); 944 vw; 936 vw; 922 w; 904 w; 892 w; 869 vw; 842 w; 814 w; 793 w; 768 w; 753 m; 730 vw; 709 w; 689 w; 668 vw; 657 w; 643 w; 623 w; 614 w; 598 vw; 591 vw; 583 vw; 576 vw; 568 vw; 561 vw; 554 vw; 546 vw; 538 vw; 510 w; 502 w; 452 w; 431 w; 411 w; 403 w. ^1^H-NMR (acetone-d_6_): 7.98–7.94 (m, 2H, o-C_6_H_5_); 7.39–7.30 (m, 1.97H, m-C_6_H_5_); 7.23–7.15 (m, 0.96H, p-C_6_H_5_); 3.12 (m, 1.19H, HC-C=O); 2.45 (m, 3.27H, CH_3_), 1.90–1.09 (m, 11.45H, cyclohexyl).

**Synthesis of [La(Q^CH^)_3_(H_2_O)(EtOH)] (7), [Gd(Q^CH^)_3_(H_2_O)] (8), and [Lu(Q^CH^)_3_(H_2_O)](9)**

Ligand **HQ^CH^**, (**1**) (0.341 g, 1.2 mmol) was dissolved in 10 mL of EtOH (96%) at 45 °C and 1.2 mL of 1 M ethanolic NaOH solution (1.2 mmol) was added dropwise with vigorous magnetic stirring. The pH of resulting solution was checked by universal indicator: it was almost neutral (pH~7), and the solution was stirred for additional 5 min. Then, the solution of corresponding hydrated chloride (0.4 mmol, 0.149 g of LaCl_3_∙7H_2_O, 0.149 g of GdCl_3_∙6H_2_O, or 0.156 g of LuCl_3_∙6H_2_O) in 3 mL of hot EtOH was slowly added and the mixture was stirred in a closed vial for additional 3 h at 45 °C. The solution become cloudy and heavy precipitate gradually formed. The suspension was cooled and the precipitate was filtered off, washed successively with 10 mL of 50% aqueous EtOH, 10 mL of deionized water, and 10 mL of hexane, and dried at 40 °C and 0.1 torr to a constant weight.

**Tris-(4-(cyclohexylcarbonyl)-5-methyl-2-phenyl-2,4-dihydro-3*H*-pyrazol-3-onato)(aqua)(ethanolo) lanthanum (III), [La(Q^CH^)_3_(H_2_O)(EtOH)] (7)**

White powder. Yield is 0.282 g (67%). Anal. calcd. for C_53_H_65_LaN_6_O_8_ (1053.02): C, 60.45; H, 6.22; N, 7.98; La, 13.19; found: C, 60.53; H, 6.29; N, 8.11; La, 13.27%. IR: 3654 vw; 3421 w ν_s_ H_2_O; 2929 m ν_as_ CH (cyclohexyl); 2853 w ν_s_ CH (cyclohexyl); 1626 vs. ν(C=O); 1610 s; 1594 s ν(C=C, C=N); 1582 s; 1529 m; 1490 vs. ν(C=C, C=N); 1455 s; 1438 s; 1412 w; 1398 m; 1377 m; 1347 w; 1341 w; 1143 vw; 1099 vw; 1077 m; 1026 w; 1013 w; 980 m δ_r_(C-H cyclohexyl); 940 vw; 894 vw; 810 w; 792 w; 761 m; 693 w; 671 w; 654 w; 621 w; 613 w; 511 vw; 497 vw; 449 vw. ^1^H-NMR (acetone-d_6_): 8.13–8.06 (m, 2H, o-C_6_H_5_); 7.32–7.18 (m, 1.97H, m-C_6_H_5_); 7.16–6.97 (m, 0.96H, p-C_6_H_5_); 2.93 (m, 1.31H, HC-C=O); 2.35 (m, 3.44H, CH_3_), 1.81–1.06 (m, 11.79H, cyclohexyl).

**Tris-(4-(cyclohexylcarbonyl)-5-methyl-2-phenyl-2,4-dihydro-3*H*-pyrazol-3-onato)(aqua) gadolinium (III), [Gd(Q^CH^)_3_(H_2_O)](8)**

White powder. Yield is 0.301 g (73%). Anal. calcd. for C_51_H_59_GdN_6_O_7_ (1025.30): C, 59.74, H, 5.80, N, 8.20, Gd,15.34; found: C,60.07, H, 5.85, N, 8.33, Gd, 15.49%. IR: 3060 m; 2930 s ν_as_ CH (cyclohexyl); 2852 m ν_s_ CH (cyclohexyl); 2669 w; 1934 vw; 1874 vw; 1805 vw; 1649 s ν(H_2_O); 1612 vs. ν(C=O); 1594 vs. ν(C=C, C=N); 1582 s; 1533 s; 1499 vs. ν(C=C, C=N); 1483 vs. ν(C=C, C=N); 1463 s; 1452 s; 1439 s; 1400 m; 1375 s; 1350 m; 1325 m; 1236 w; 1203 vw; 1176 w; 1130 w; 1080 s; 1028 m; 1011 m; 1000 w; 980 s δ_r_(C-H cyclohexyl); 922 vw; 903 w; 892 w; 868 vw; 844 w; 812 m; 792 w; 768 m; 754 m; 704 w; 689 m; 655 m; 643 w; 621 m; 614 w; 533 vw; 509 w; 497 w; 449 w; 417 w.

**Tris-(4-(cyclohexylcarbonyl)-5-methyl-2-phenyl-2,4-dihydro-3*H*-pyrazol-3-onato)(aqua) lutetium (III), [Lu(Q^CH^)_3_(H_2_O)] (9)**

White powder. Yield is 0.329 g (78%). Anal. calcd. for C_51_H_59_LuN_6_O_7_ (1043.03): C, 58.73, H, 5.70; N, 8.06; Lu, 16.78; found: C, 58.81, H, 5.76; N, 8.14%; Lu, 16.91%. IR: 3437 vw ν_s_ H_2_O; 3060 vw; 2930 m ν_as_ CH (cyclohexyl); 2852 w ν_s_ CH (cyclohexyl); 2794 vw; 2668 vw; 1936 vw; 1653 m δ(H_2_O); 1615 vs. ν(C=O); 1595 s; ν(C=C, C=N) 1583 m; 1534 m; 1501 s ν(C=C, C=N); 1486 s; 1464 m; 1451 m; 1440 m; 1401 w; 1379 m; 1349 vw; 1325 vw; 1238 vw; 1177 vw; 1131 vw; 1082 m; 1029 w; 1012 w; 999 vw; 981 m δ_r_(C-H cyclohexyl); 922 vw; 905 vw; 892 vw; 844 vw; 814 w; 792 w; 783 vw; 769 w; 753 m; 707 vw; 700 vw; 689 w; 668 vw; 656 w; 644 vw; 622 w; 615 w; 509 w; 501 vw; 450 w; 425 vw; 420 vw. ^1^H-NMR (DMSO-d^6^): 8.57–8.51 (m, 2H, o-C_6_H_5_); 7.78–7.62 (m, 2.05H, m-C_6_H_5_); 7.55–7.44 (m, 1.04H, p-C_6_H_5_); 3.38 (m, 1.02H, HC-C=O); 2.80 (m, 3.03H, CH_3_), 2.27–1.53 (m, 11.12H, cyclohexyl).

## 5. Conclusions

The influence of the type of central ion on the photophysical properties of the **HQ^CH^** ligand was thoroughly investigated. We found that coordinating the **HQ^CH^** by different trivalent metal ions increased the energies of both the first excited singlet and triplet states by 1500 cm^−1^, except for the [Sc(Q^CH^)_3_(H_2_O)]_n_ complex, which showed a triplet increase of 600 cm^−1^. However, the singlet–triplet energy gap slightly oscillated around 3900 cm^−1^ for all the compounds. According to the Fermi golden rule, the intersystem crossing rate (k_isc_) is proportional to the square of the energy transfer matrix element and inversely proportional to the energy gap. However, replacement of the Al^3+^ ion by a heavier one, such as Ga^3+^ or In^3+^, increased k_isc_ from 6.3 × 10^7^ s^−1^ to 8.9 × 10^7^ s^−1^. Notably, [Gd(Q^CH^)_3_(H_2_O)] was the only complex that demonstrated room temperature phosphorescence with approximately the same k_isc_ rate as **HQ^CH^**. Therefore, we conclude that this phenomenon is not related to spin–orbit coupling enhancement, but rather to the paramagnetic properties of Gd^3+^ ion.

In this study, we demonstrated that **HQ^CH^** predominantly emits from the T_1_ state (phosphorescence). Trivalent metal ions fully suppress the ligand’s phosphorescence, except for Gd, which is due to the paramagnetic properties of the Gd^3+^ ion.

We observed a significant increase in the quantum yield values up to 39 times for the complexes in comparison with **HQ^CH^**. We established that the coordination compounds of lanthanide ions have the highest photoluminescence efficiencies, which are 19.5% for [La(Q^CH^)_3_(H_2_O)(EtOH)] and 19.0% for [Gd(Q^CH^)_3_(H_2_O)].

Thus, this paper shows that the nature of the non-luminescent cation has a dramatic effect on the luminescence features of acylpyrazolonate complexes. By varying the cation, one can control the nature of the emission, the energies of the singlet and triplet states, and the lifetime of the excited states. The regularities shown in this work will be useful for the design of a new generation of WOLED devices.

## Figures and Tables

**Table 1:** Crystal data and refinement parameters for [Al(Q^CH^)_3_], [Ga(Q^CH^)_3_], and [In(Q^CH^)_3_] (**2**–**4**).

Parameter	[Al(Q^CH^)_3_]	[Ga(Q^CH^)_3_]	[In(Q^CH^)_3_]
Molecular Formula	C_51_H_56_AlN_6_O_6_	C_51_H_56_GaN_6_O_6_	C_51_H_56_InN_6_O_6_
M	875.99	918.73	964.84
Temperature, K	100(2)	110(2)	100(2)
System	Monoclinic	Monoclinic	Triclinic
Space group	P*2*_1_*/c*	P*2*_1_*/c*	P*-1*
a, Å	17.9838(8)	17.865(4)	13.2087(14)
b, Å	13.0521(6)	12.918(3)	20.270(2)
c, Å	19.8671(9)	20.037(4)	20.561(3)
α, deg.	90	90	110.542(5)
β, deg.	92.303(2)	92.95(3)	93.380(5)
γ, deg.	90	90	108.234(4)
V, Å^3^	4659.6(4)	4618.3(16)	4807.3(10)
Z	4	4	4
ρ_calc_, g/cm^3^	1.249	1.321	1.333
μ(Mo*K*_α_), mm^−1^	0.100	0.653	0.546
*F*(000)	1860	1932	2008
θmin–θmax, deg.	1.87–25.00	2.04–26.00	1.64–26.00
Number of measured reflections	32,091	31,594	54,548
Number of unique reflections (R_int_)	8200 (0.0742)	9059 (0.0633)	18,843 (0.0792)
Number of reflections with *I* > 2σ(*I*)	5427	6154	13,863
Number of refined parameters	568	556	1159
R-factors (*I* > 2σ(*I*))	R_1_ = 0.1117, ωR_2_ = 0.2928	R_1_ = 0.1049, ωR_2_ = 0.2757	R_1_ = 0.0627, ωR_2_ = 0.1397
R-factors (all reflections)	R_1_ = 0.1499, ωR_2_ = 0.3274	R_1_ = 0.1414, ωR_2_ = 0.3028	R_1_ = 0.0914, ωR_2_ = 0.1566
GOOF	1.026	1.052	1.021
Δρ_max_/Δρ_min_, e/Å^3^	1.534/−0.557	1.743/−0.941	1.999/−0.883

**Table 2:** Crystal data and refinement parameters for [Sc(Q^CH^)_3_(DMSO)], [La(Q^CH^)_3_(H_2_O)(EtOH)]∙(EtOH), [Gd(Q^CH^)_3_(H_2_O)], and [Lu(Q^CH^)_3_(DMSO)] (**6**-**9**).

Parameter	[Sc(Q^CH^)_3_(DMSO)]	[La(Q^CH^)_3_(H_2_O) (EtOH)]∙(EtOH)	[Gd(Q^CH^)_3_(H_2_O)]	[Lu(Q^CH^)_3_(DMSO)]
Molecular Formula	C_53_H_63_ScSN_6_O_7_	C_55_H_71_LaN_6_O_9_	C_51_H_59_GdN_6_O_7_	C_53_H_63_LuSN_6_O_7_
M	973.11	1099.09	1025.29	1103.12
Temperature, K	293(2)	296(2)	110(2)	293(2)
System	Triclinic	Monoclinic	Triclinic	Triclinic
Space group	P-1	C2/c	P-1	P-1
a, Å	12.8644(8)	18.4623(13)	9.8233(8)	12.9949(13)
b, Å	14.7619(9)	21.8926(16)	13.9771(11)	14.8199(14)
c, Å	15.1365(12)	27.0312(18)	18.7740(14)	15.271(2)
α, deg.	105.270(3)	90	76.367(3)	105.903(4)
β, deg.	113.938(2)	103.774(2)	81.709(4)	113.978(4)
γ, deg.	90.810(2)	90	77.638(3)	90.267(3)
V, Å^3^	2510.5(3)	10,611.5(13)	2435.0(3)	2561.7(5)
Z	2	8	2	2
ρ_calc_, g/cm^3^	1.287	1.376	1.398	1.430
μ(Mo*K*_α_), mm^−1^	0.246	0.866	1.418	2.024
*F*(000)	1032	4576	1054	1132
θmin–θmax, deg.	2.40–28.28	2.27–26.02	1.69–28.00	2.10–28.28
Number of measured reflections	27,067	41,943	40,875	25,256
Number of unique reflections (R_int_)	12,396 (0.0951)	10,447 (0.1536)	11,740 (0.1026)	12,647 (0.0283)
Number of reflections with *I* > 2σ(*I*)	6081	6198	9736	11,216
Number of refined parameters	618	631	592	618
R-factors (*I* > 2σ(*I*))	R_1_ = 0.0857, ωR_2_ = 0.1572	R_1_ = 0.0924, ωR_2_ = 0.2088	R_1_ = 0.0496, ωR_2_ = 0.1040	R_1_ = 0.0291, ωR_2_ = 0.0550
R-factors (all reflections)	R_1_ = 0.1775, ωR_2_ = 0.1934	R_1_ = 0.1543, ωR_2_ = 0.2415	R_1_ = 0.0640, ωR_2_ = 0.1094	R_1_ = 0.0365, ωR_2_ = 0.0576
GOOF	1.000	1.018	1.026	1.020
Δρmax/Δρmin, e/Å^3^	0.415/−0.698	1.078/−2.214	2.177/−1.416	0.618/−0.670

**Table 3:** Photophysical parameters for all the compounds.

Compound	λ_abs_	λ_em_	λ_exc_	E(S_1_) × 10^3^	E(T_1_) × 10^3^	Energy Gap × 10^3^	τ_obs_	Φ
	nm	nm	nm	cm^−1^	cm^−1^	cm^−1^	ns	%
**HQ^CH^**	267	530	361	26.0	22.1	3.9	7.4 ± 0.1	0.5 ± 0.1
**Sc**	271	430	338	27.3	22.7	4.5	2.7 ± 0.1 6.3 ± 0.1	10.6 ± 0.1
**La**	269	436	339	27.4	23.6	3.8	3.4 ± 0.1 8.7 ± 0.1	19.5 ± 0.1
**Gd**	265	490	343	27.7	23.6	4.1	(22.3 ± 0.2) × 10^3^ (36.5 ± 0.3) × 10^3^	19.0 ± 0.1
**Lu**	276	428	323	28.0	23.7	4.3	1.7 ± 0.1 4.1 ± 0.1	6.5 ± 0.1
**Al**	277	458	345	27.6	23.7	3.8	9.8 ± 0.1	16.9 ± 0.1
**Ga**	278	463	342	27.0	23.5	3.5	6.3 ± 0.1	6.6 ± 0.1
**In**	278	464	340	27.6	23.6	4.0	4.2 ± 0.1	3.0 ± 0.1

**Table 4:** Radiative (k_rad_) and non-radiative (k_nrad,_ k_isc_) rate constants in s^−1^ for the **Al**, **In**, **Ga**, and **Gd** compounds (**2**-**4** and **8**) and free ligand (**1**).

Compound	k_rad_ × 10^7^	k_nrad_ × 10^8^	^a^ k_isc_ × 10^7^	^b^ k_isc_ × 10^7^
**HQ^CH^**	0.1	1.4	13.4	13.3
**Al**	1.7	1.0	8.5	6.3
**Ga**	1.0	1.6	14.8	8.6
**In**	0.7	2.4	23.3	8.9
**Gd**	-	-	12.5	13.8

^a^ Measured at 300 K, ^b^ measured at 77 K.

## Data Availability

Data available upon request.

## Supplementary Materials

The supporting information can be downloaded at https://www.mdpi.com/article/10.3390/ijms24098131/s1.
