# Supplementary material for: Dynamics of the Ligand Excited States Relaxation in Novel β-Diketonates of Non-Luminescent Trivalent Metal Ions
Source: Int J Mol Sci. 2023 May 1;24(9):8131. doi: 10.3390/ijms24098131 (PMC10179517; doi:10.3390/ijms24098131)
Supplement: Supplementary file 1 [file ijms-24-08131-s001.zip › ijms-2352732-supplementary.pdf]

Supporting information

Dynamics of the ligand excited states relaxation in novel β -diketonates of non-luminescent trivalent metal ions

Trofim Polikovskiy[1], Vladislav Korshunov [1], Victoria Gontcharenko [1,5], Michal Kiskin[2], Yuriy A. Belousov[1,3], Claudio Pettinari [4] and Ilya Taydakov [1,*]

1 P. N. Lebedev Physical Institute of the Russian Academy of Sciences, 53 Leninskiy 1. Prospect, 119991 Moscow, Russia

2 Kurnakov Institute of General and Inorganic Chemistry, Russian Academy of Sciences, 119991 Moscow, Russia

3 Chemistry Department, M.V. Lomonosov Moscow State University, Leninskie Gory Str, Building 1/3, Moscow 119991, Russia

4 School of Pharmacy, Chemistry Interdisciplinary Project (ChIP), University of Camerino, Via Madonna delle Carceri, 62032 Camerino, Italy

5 National Research University Higher School of Economics, Department of Chemistry, 20 Miasnitskaya Str., Moscow 101000, Russia

* Correspondence: ivt@lebedev.ru;

X-ray structure determination

Experimental

Single-crystal X-ray diffraction analysis of [Al(Q^{CH})$_3$], [Ga(Q^{CH})$_3$], [In(Q^{CH})$_3$], [Sc(Q^{CH})$_3$(DMSO)], [La(Q^{CH})$_3$(H_2O)(EtOH)]·(EtOH), [Gd(Q^{CH})$_3$(H_2O)] and [Lu(Q^{CH})$_3$(DMSO)] was carried out on a Bruker D8 Quest diffractometer (MoKα radiation, ω and φ-scan mode). The structures were solved with direct methods and refined by least-squares method in the full-matrix anisotropic approximation on F^2. High reported values of R_1-factors for [Al(QCH)3] and [Ga(QCH)3] are due to their weak scattering of X-ray caused by disorder of cyclohexyl substituents. All hydrogen atoms were located in calculated positions and refined within riding model. All calculations were performed using the SHELXTL [1, 2] and Olex2 [3] software packages. Atomic coordinates, bond lengths, angles, and thermal parameters have been deposited at the Cambridge Crystallographic Data Centre with deposition numbers—CCDC 2208569-2208571, 2215463, 2215494, 2215723, 2215486, which are available free of charge at www.ccdc.cam.ac.uk.

[1] Sheldrick G.M. // Acta Crystallogr. C. 2015. V. 71. P. 3.

[2] Sheldrick G.M. // Acta Crystallogr. A. 2015. V. 71. P. 3.

[3] Dolomanov O.V., Bourhis L.J., Gildea R.J. et al. // J. Appl. Crystallogr. 2009. V. 42. P. 339.

Table S1. *List of metal-oxygen bond lengths in [Al(Q^{CH})$_3$], [Ga(Q^{CH})$_3$] and [In(Q^{CH})$_3$] complexes and their mean value.*

Bond	**[Al(Q^{CH})$_3$]**	**[Ga(Q^{CH})$_3$]**	**[In(Q^{CH})$_3$]**
M-O1, Å	1.861(4)	1.932(4)	2.113(3)<br>2.119(3)
M-O2, Å	1.894(4)	1.967(4)	2.149(3)<br>2.116(3)
M-O3, Å	1.873(4)	1.937(4)	2.129(3)<br>2.103(3)
M-O4, Å	1.905(4)	1.943(5)	2.130(3)<br>2.150(3)
M-O5, Å	1.881(4)	1.922(5)	2.117(3)<br>2.120(3)
M-O6, Å	1.878(4)	1.973(5)	2.132(3)<br>2.136(3)
mean M-O, Å	1.88	1.95	2.13

Table S2. *List of metal-oxygen bond lengths in [Sc(Q^{CH})$_3$(DMSO)], [La(Q^{CH})$_3$(H_2O)(EtOH)]·(EtOH), [Gd(Q^{CH})$_3$(H_2O)] and [Lu(Q^{CH})$_3$(DMSO)] complexes and their mean value.*

Bond	**[Sc(Q^{CH})$_3$(DMSO)]**	**[La(Q^{CH})$_3$(H_2O)(EtOH)]·(EtOH)**	**[Gd(Q^{CH})$_3$(H_2O)]**	**[Lu(Q^{CH})$_3$(DMSO)]**
M-O1, Å	2.093(3)	2.433(6)	2.325(3)	2.1874(16)
M-O2, Å	2.192(3)	2.524(6)	2.336(3)	2.2802(17)
M-O3, Å	2.067(3)	2.445(6)	2.277(3)	2.2017(16)
M-O4, Å	2.233(3)	2.466(6)	2.325(3)	2.2948(17)
M-O5, Å	2.096(3)	2.413(6)	2.290(3)	2.2024(17)
M-O6, Å	2.167(3)	2.523(6)	2.349(3)	2.2512(16)
M-O7, Å	2.206(3)	2.577(6)	2.336(3)	2.2875(17)
M-O8, Å	-	2.601(8)	-	-
mean M-O, Å	2.15	2.50	2.32	2.244

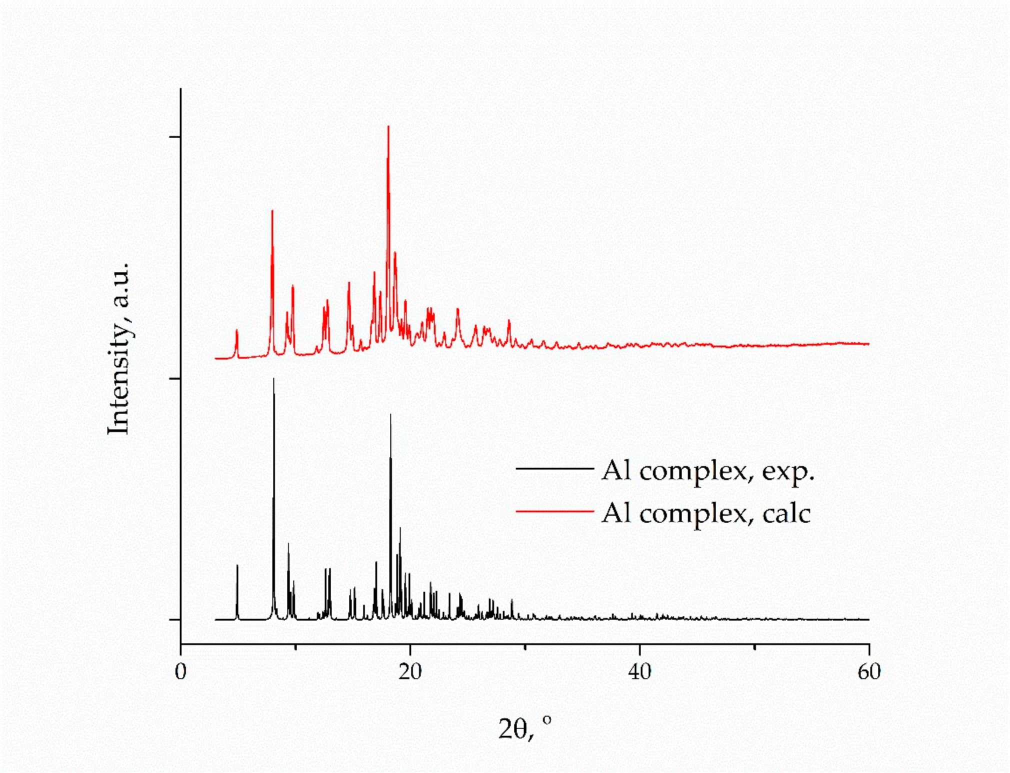

Figure S1. Comparison of the experimental and theoretical pattern of PXRD for the [Al$Q^{CH}{}_3$] complex.

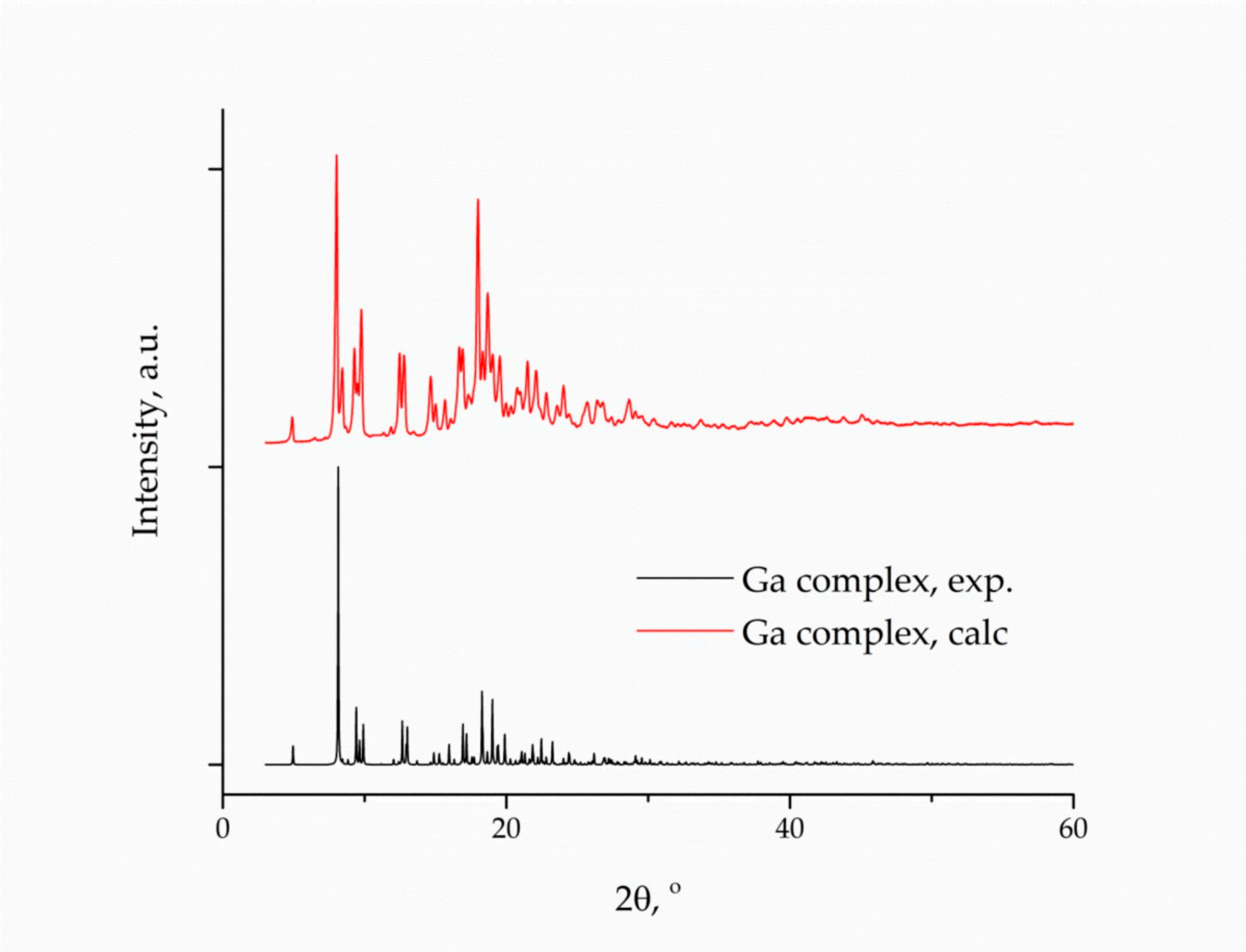

Figure S2. Comparison of the experimental and theoretical pattern of PXRD for the [GaQ^{CH_3}] complex.

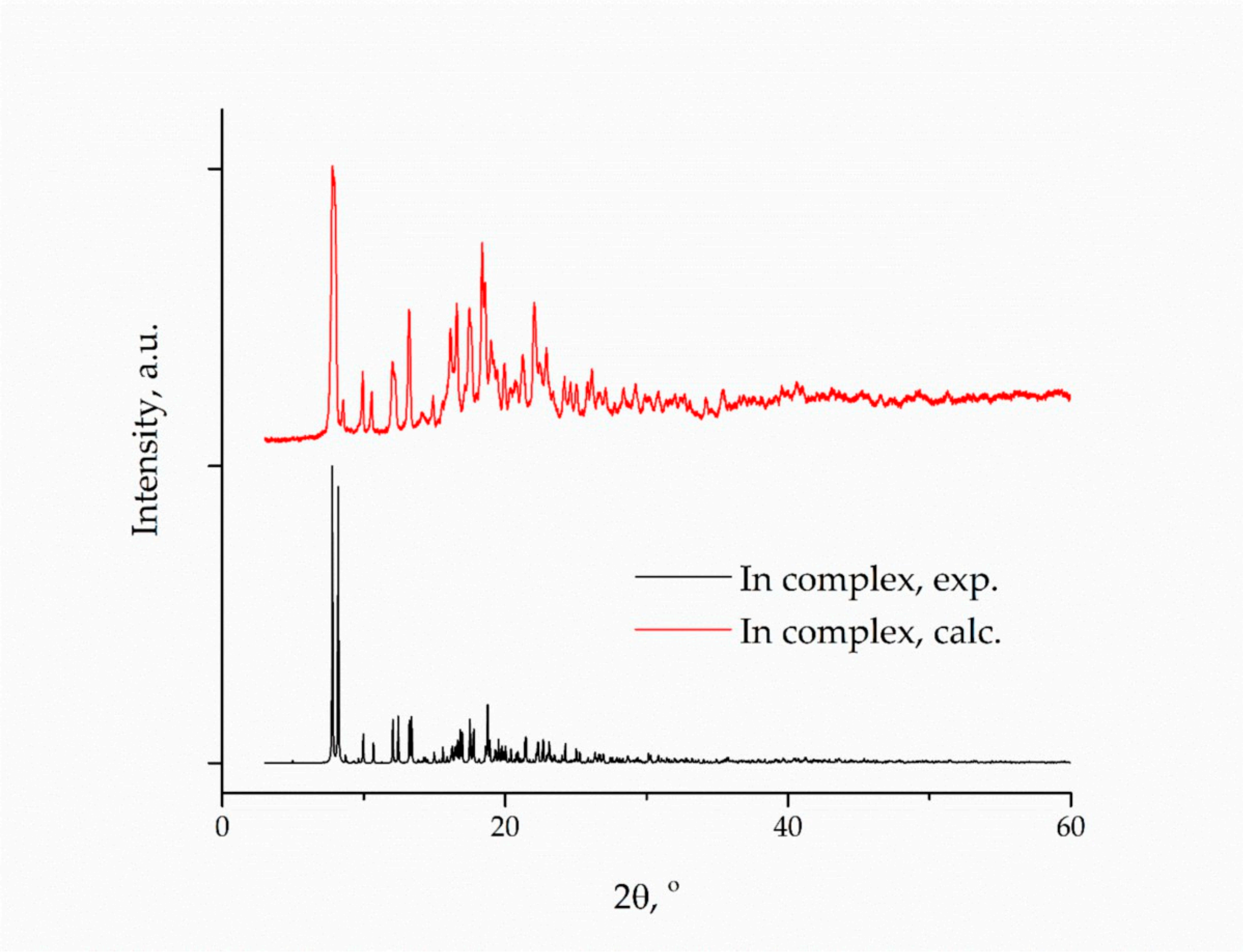

Figure S3. Comparison of the experimental and theoretical pattern of PXRD for the [In(Q^{CH})$_3$] complex.

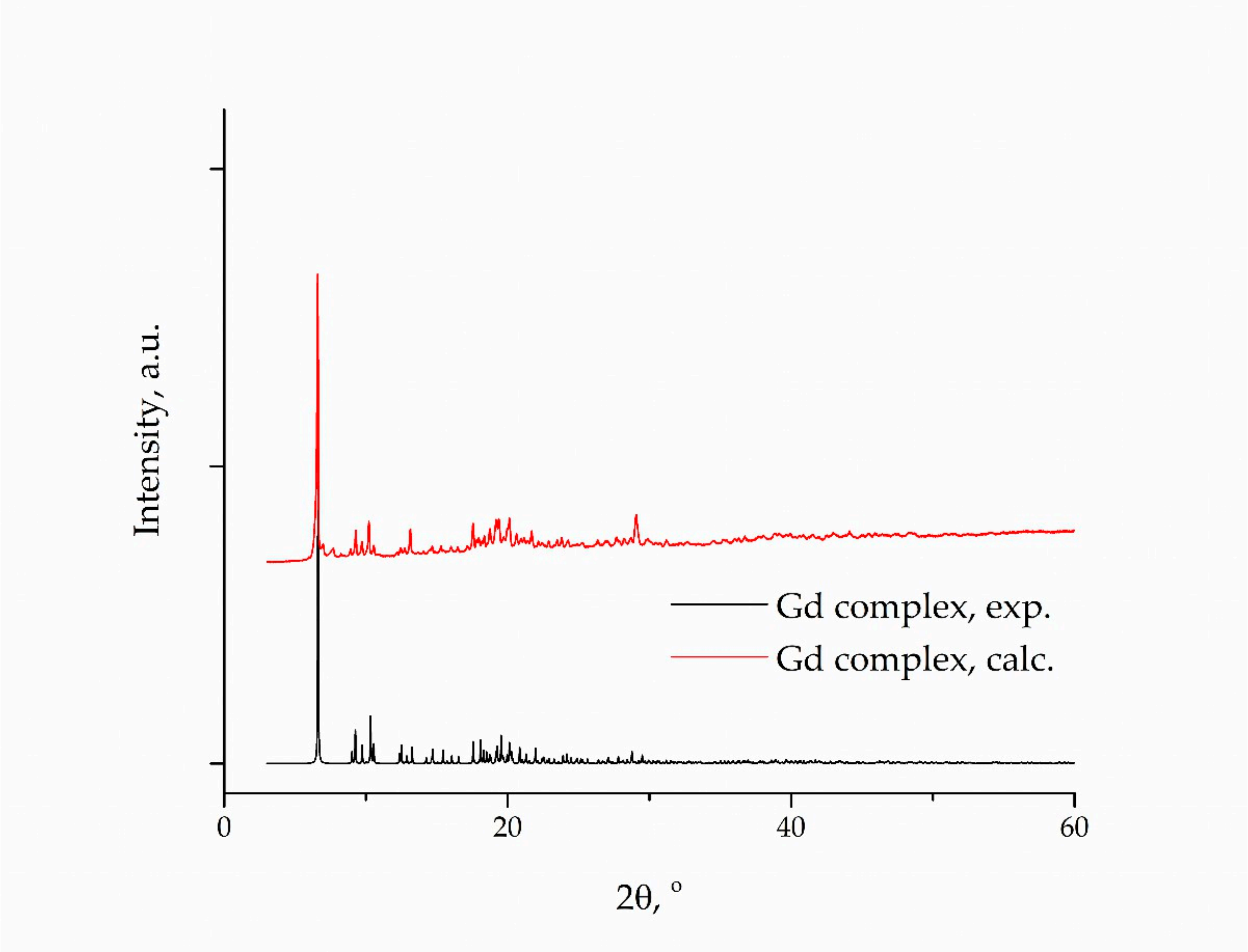

Figure S4. Comparison of the experimental and theoretical pattern of PXRD for the $[Gd(Q^{CH})_3(H_2O)]$ complex.

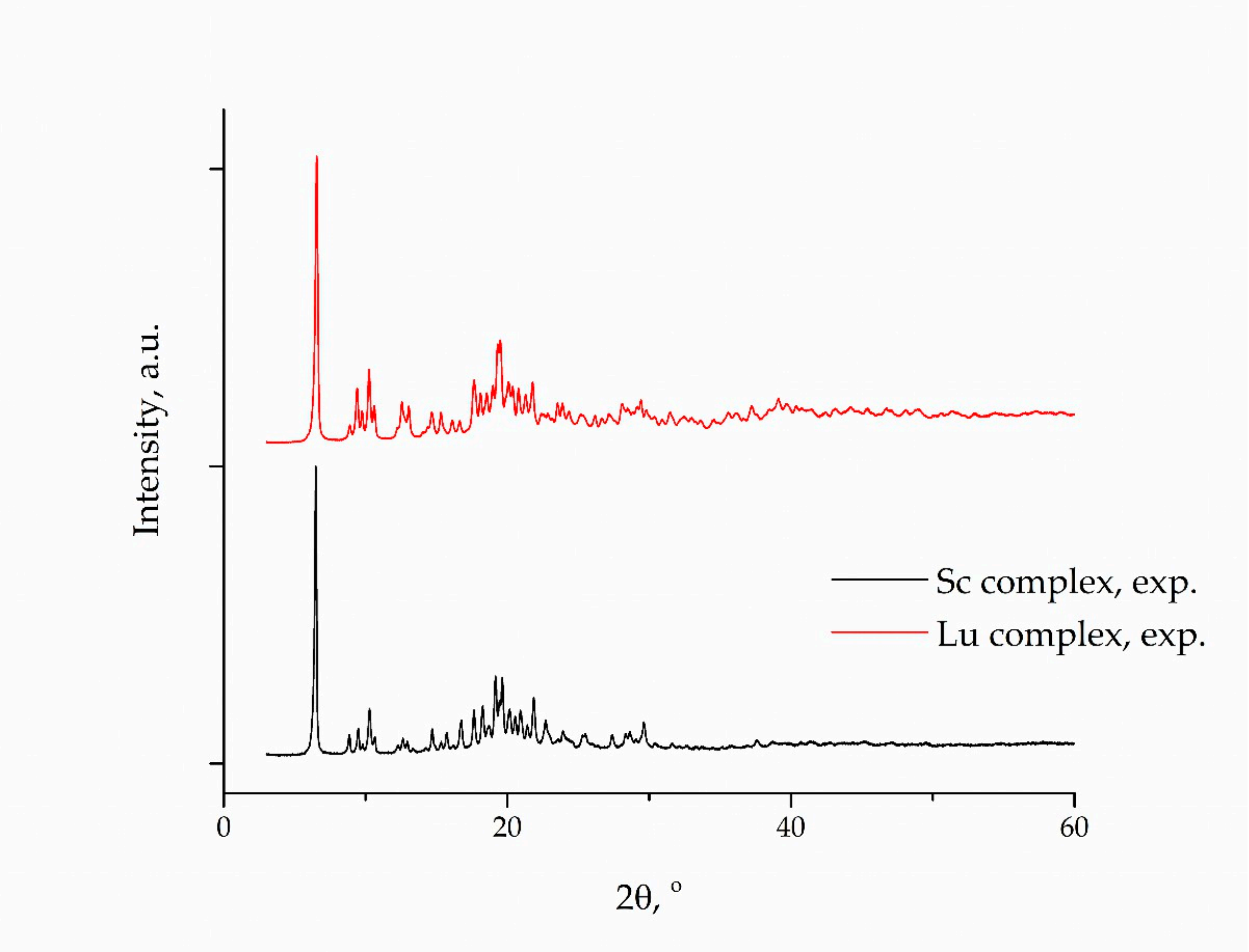

Figure S5. Comparison of experimental PXRD data for [Sc(Q^{CH})$_3$(H_2O)] and [Lu(Q^{CH})$_3$(H_2O)] complexes.

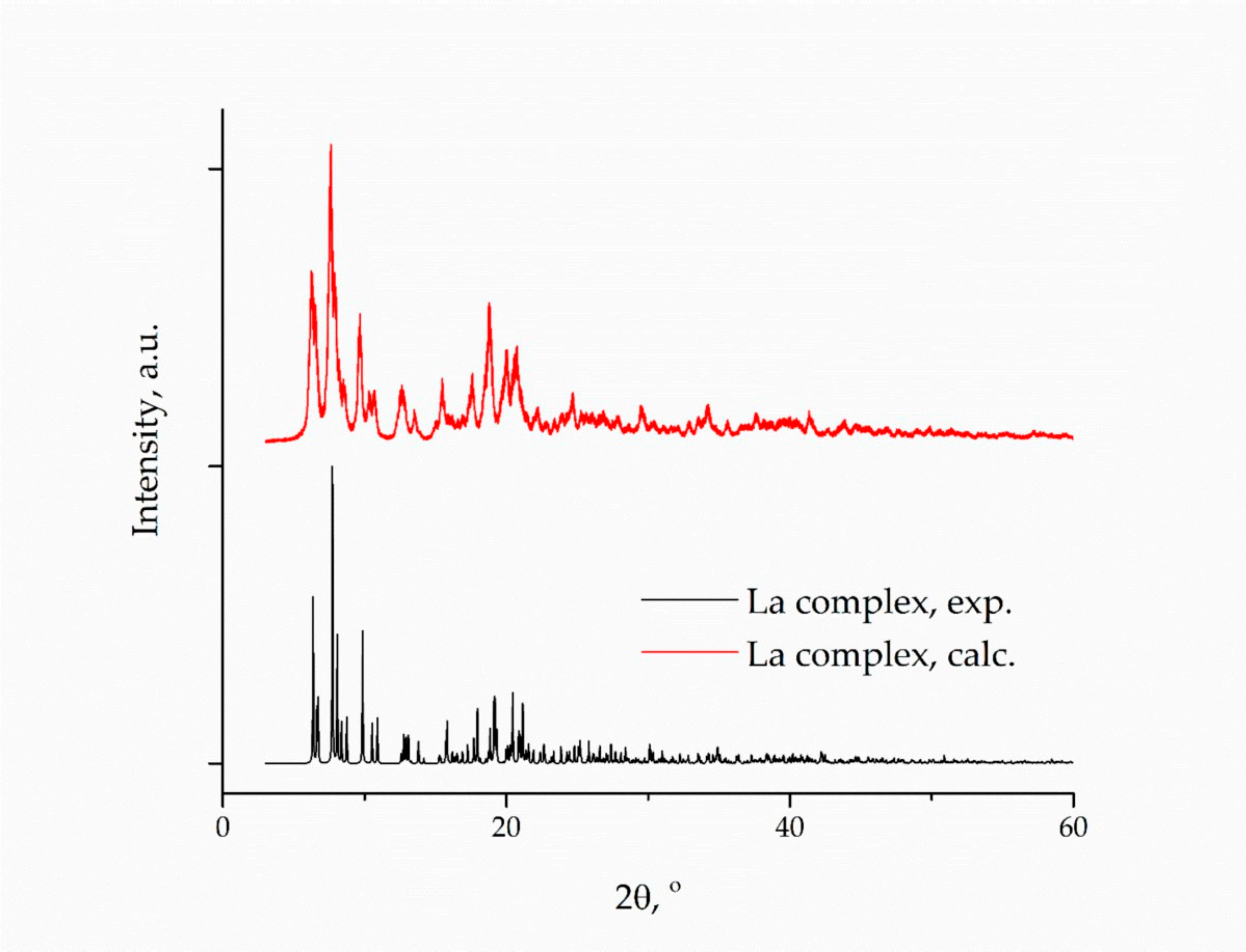

Figure S6. Comparison of the experimental and theoretical pattern of PXRD for the $[La(Q^{CH})_3(H_2O)(EtOH)]\cdot(EtOH)$ complex.

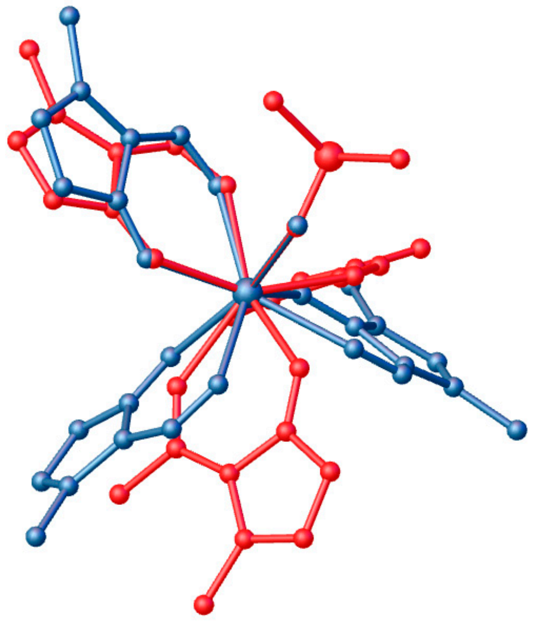

Figure S7. View of the overlayed fragments of [Lu(Q^{CH})$_3$(DMSO)] (red) and [Gd(Q^{CH})$_3$(H_2O)] (blue). Hydrogen atoms, phenyl and cyclohexyl fragments are not shown for clarity, thermal ellipsoids are not illustrated.

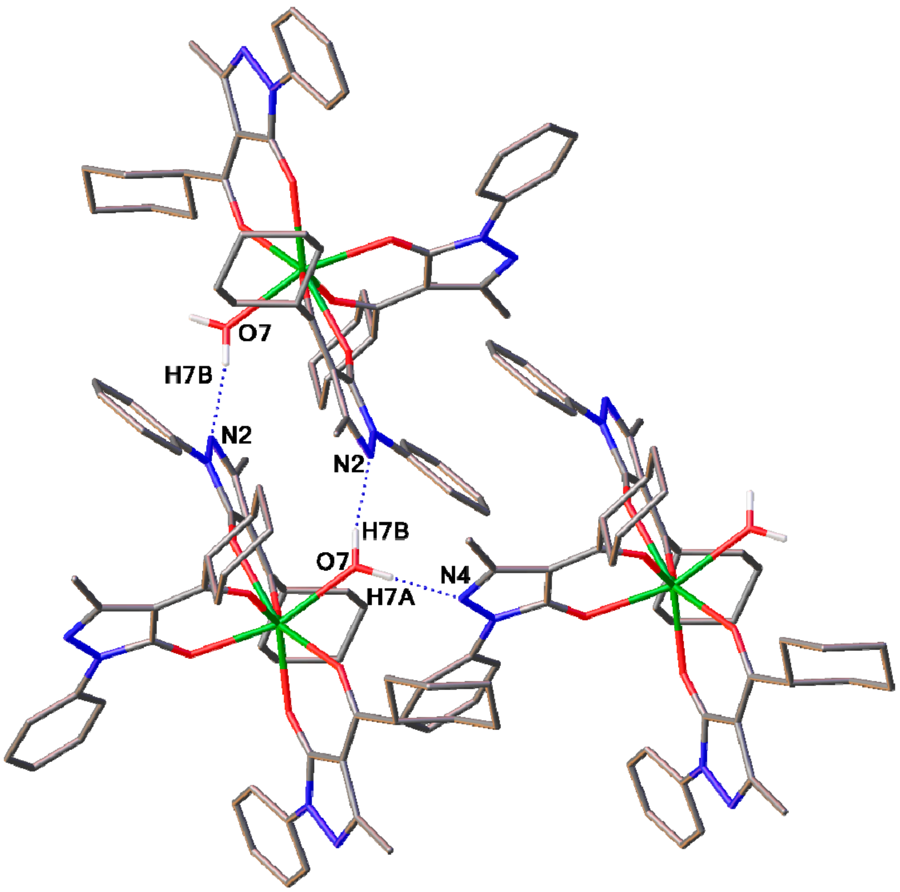

Figure S8. View of the crystal packing of [Gd(Q^{CH})$_3$(H_2O)]. Hydrogen atoms not involved in intermolecular interactions are not shown for clarity, thermal ellipsoids are not illustrated. Hydrogen bonds are shown with dotted lines.

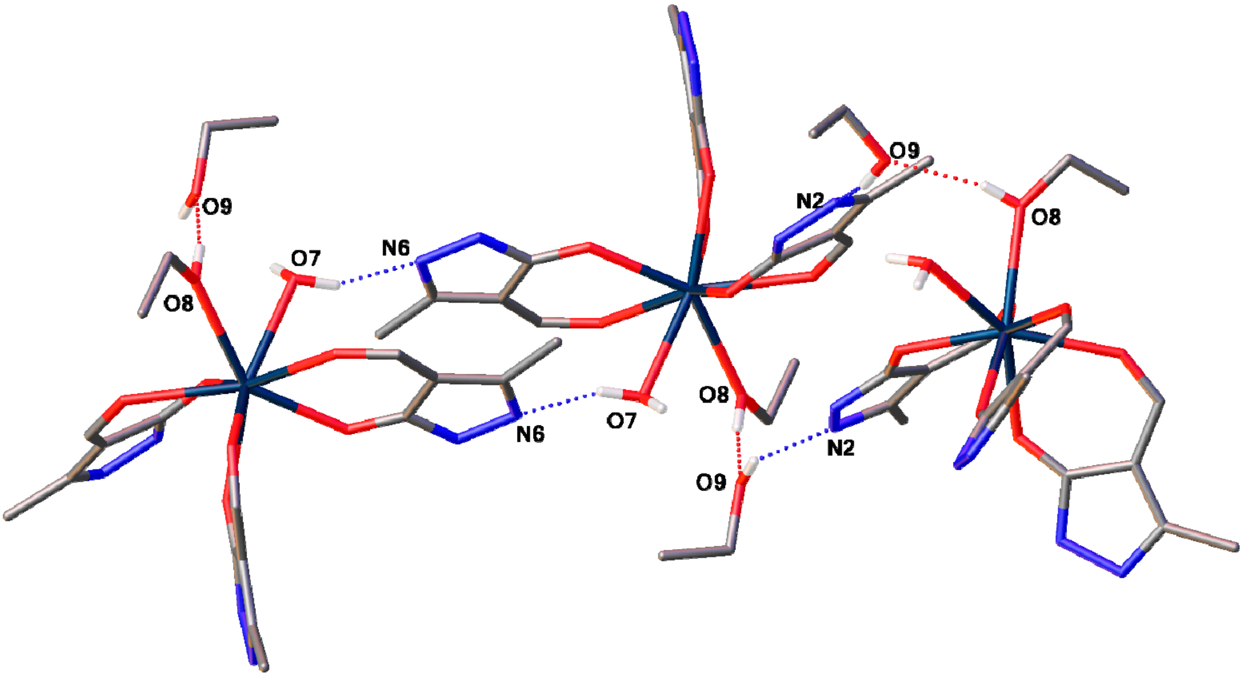

Figure S9. View of the crystal packing of [La(Q^{CH})$_3$(H_2O)(EtOH)]·(EtOH). Hydrogen atoms not involved in intermolecular interactions are not shown for clarity, phenyl and cyclohexyl fragments as well as thermal ellipsoids are not illustrated. Hydrogen bonds are shown with dotted lines.

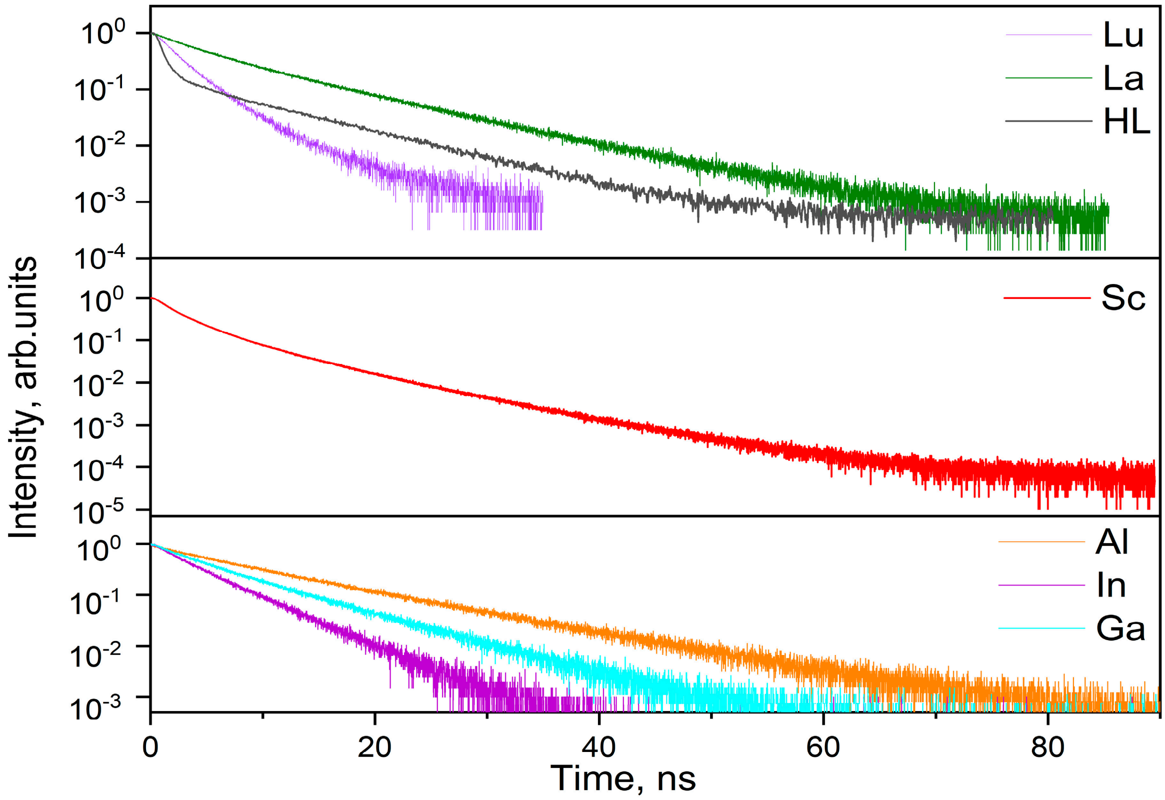

Figure S10. Photoluminescence decays for all the complexes and free ligand (**1**) under optical excitation of 340 nm registered at the PL maxima respectively.

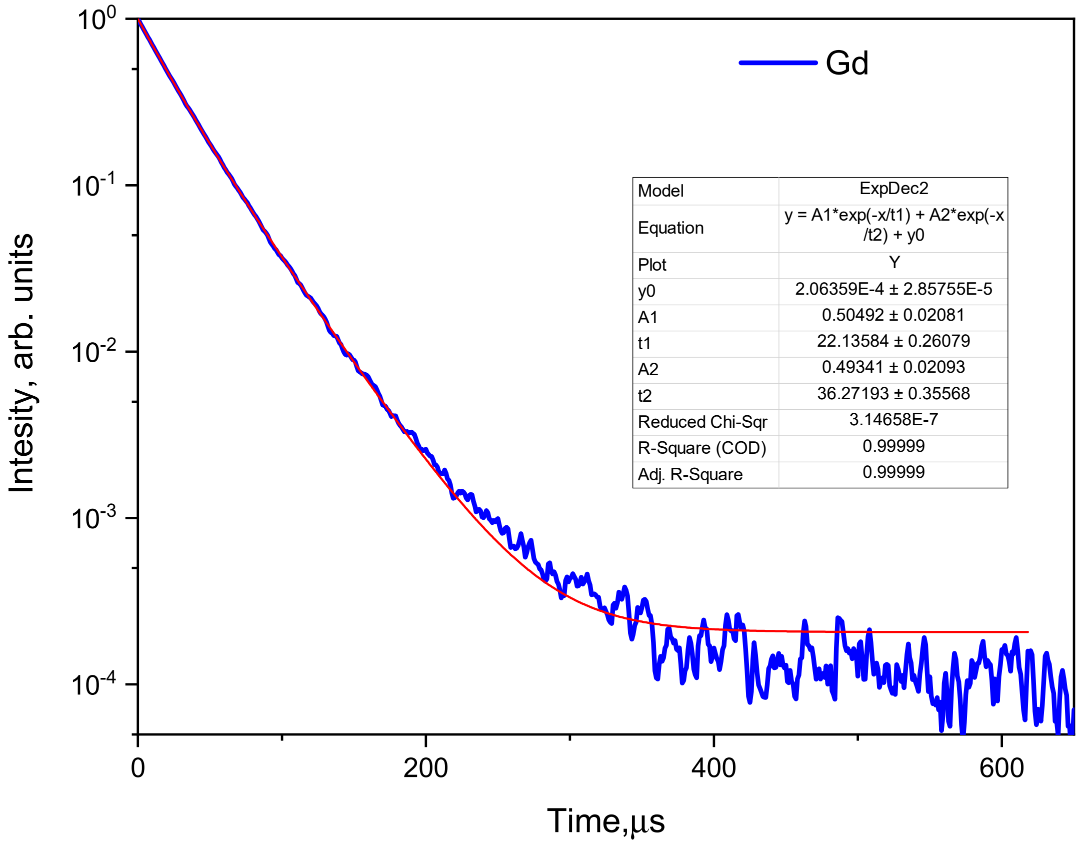

Figure S11. Photoluminescence decay for Gd complex **8** under optical excitation of 340 nm registered at the PL maximum

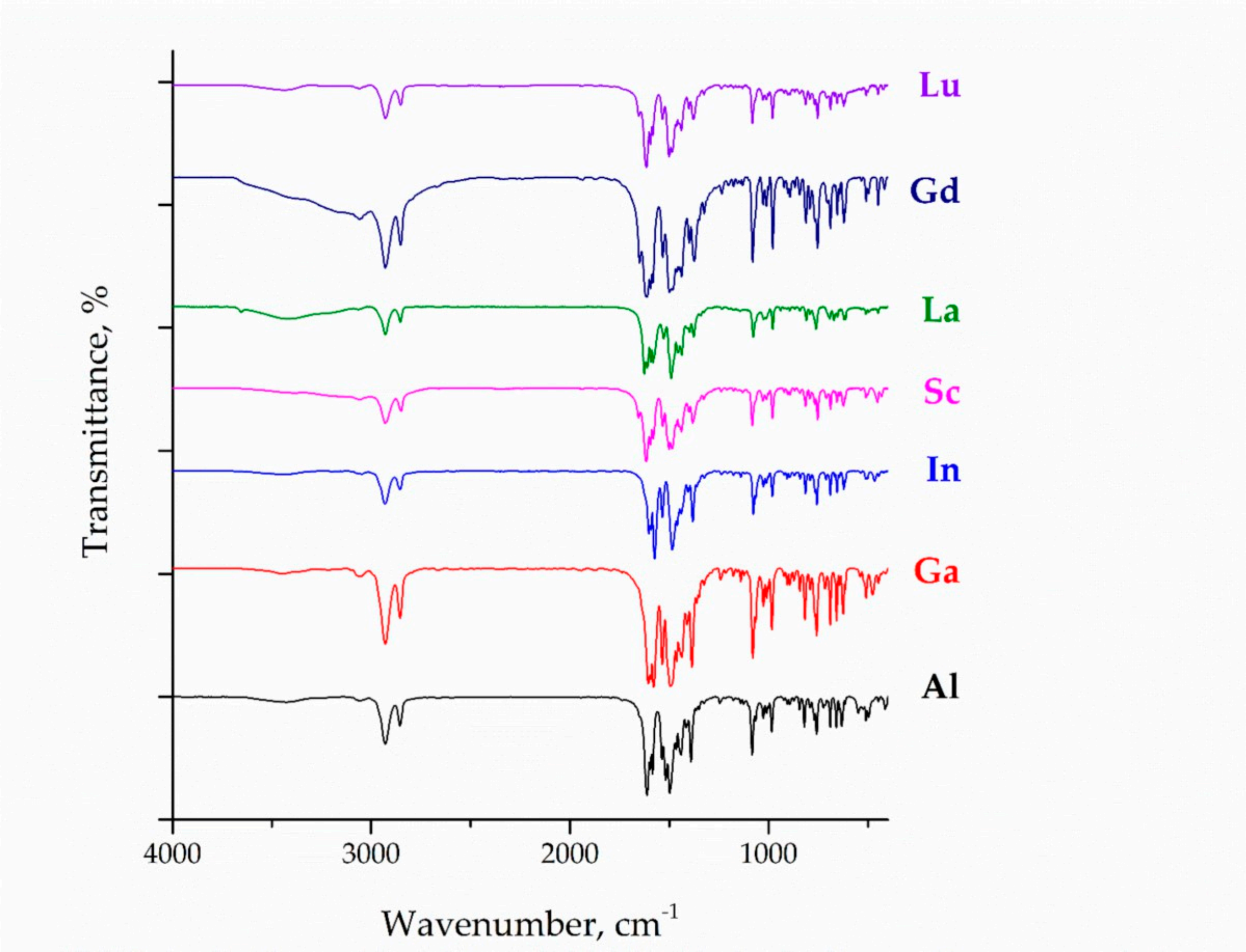

Figure S12. Infrared spectra of complexes.
